# What are Effective Strategies to Reduce Low-Value Care? An Analysis of 121 Randomized Deimplementation Studies

**DOI:** 10.1097/JHQ.0000000000000392

**Published:** 2023-06-29

**Authors:** Pauline Heus, Simone A. van Dulmen, Jan-Willem Weenink, Christiana A. Naaktgeboren, Toshihiko Takada, Eva W. Verkerk, Isabelle Kamm, Maarten J. van der Laan, Lotty Hooft, Rudolf B. Kool

**Keywords:** low-value care, overuse, deimplementation, quality improvement, systematic review

## Abstract

**Background:**

Low-value care is healthcare leading to no or little clinical benefit for the patient. The best (combinations of) interventions to reduce low-value care are unclear.

**Purpose:**

To provide an overview of randomized controlled trials (RCTs) evaluating deimplementation strategies, to quantify the effectiveness and describe different combinations of strategies.

**Methods:**

Analysis of 121 RCTs (1990–2019) evaluating a strategy to reduce low-value care, identified by a systematic review. Deimplementation strategies were described and associations between strategy characteristics and effectiveness explored.

**Results:**

Of 109 trials comparing deimplementation to usual care, 75 (69%) reported a significant reduction of low-value healthcare practices. Seventy-three trials included in a quantitative analysis showed a median relative reduction of 17% (IQR 7%–42%). The effectiveness of deimplementation strategies was not associated with the number and types of interventions applied.

**Conclusions and Implications:**

Most deimplementation strategies achieved a considerable reduction of low-value care. We found no signs that a particular type or number of interventions works best for deimplementation. Future deimplementation studies should map relevant contextual factors, such as the workplace culture or economic factors. Interventions should be tailored to these factors and provide details regarding sustainability of the effect.

## Abstract 2

Supplemental Digital Content is Available in the Text.

## Introduction

Low-value care (LVC) is healthcare that has no or little clinical benefit for the patient, considering the costs, the risks, available alternatives, and patient preferences.^1^ Estimates of the volume of LVC range from 10% to 30%, with estimates up to 89% for specific healthcare practices.^1-6^

Low-value care and strategies to reduce it have received increasing attention. In the last decade, several initiatives have been launched that list practices that doctors and patients should question or withhold.^7-10^ Yet, raising awareness by presenting lists is not enough to reduce the use of these practices.^11,12^ Previous research showed that active dissemination strategies are more likely to be effective, rather than passive dissemination strategies, such as publishing a guideline on a website.^13,14^ It is unclear which active strategies are the best to reduce LVC, as a scoping review concluded in 2015.^15^ One of the recommendation of this scoping review was to undertake a more detailed evidence synthesis to quantify the effectiveness of so called deimplementation strategies.

In 2016, a systematic review of interventions aimed at reducing LVC indicated that multicomponent interventions are potentially more effective than single-component interventions, especially when addressing patients and clinicians.^1^ This overview was descriptive, without comparing the absolute or relative measures of the effect of deimplementation strategies. More recently, another review focused on controlled and uncontrolled studies related to deimplementation of nursing procedures.^16^ Most studies with a significant positive effect used a deimplementation strategy with an educational component (educational meetings, educational materials, educational outreach visits, or educational games) and focused their deimplementation strategy at reducing the use of restraints. Finally, in 2021 a review on the effectiveness of interventions to implement Choosing Wisely guidelines showed that multicomponent interventions targeting clinicians are the most effective types of interventions.^17^ In these previous reviews, the effect was not always quantified and also observational studies without a parallel control group were included, making it hard to draw conclusions about effectiveness of strategies.

Our aim was to provide an overview of randomized controlled trials (RCT) evaluating deimplementation strategies, to quantify the effectiveness and to describe different combinations of strategies. Our findings will contribute to the evidence-base needed for facilitating the development of effective and sustainable deimplementation strategies to improve quality of care.

## Methods

This study was performed as part of a national program aimed at reducing LVC.^18^ Within the scope of this overarching program, a systematic review of the literature on deimplementation was performed, which is used as a basis for this manuscript. The review protocol has not been registered or published. The methodology we used matches that of a systematic review: a rigorous and systematic method to synthesize the evidence on effectiveness of deimplementation, including assessment of risk of bias and a quantitative analysis of the results. The Preferred Reporting Items for Systematic reviews and Meta-Analyses (PRISMA) statement was followed.^19^

### Systematic Review

Details on the systematic review are provided in the Supplementary File A (http://links.lww.com/JHQ/A189). In total, 121 randomized controlled trials (RCTs) published between 1990 and November 2019 and studying a strategy aimed at reducing LVC were included. LVC was defined as a healthcare practice that according to the study authors was considered inappropriate. Deimplementation strategies were described according to their targets (provider, patient, organizational context, and healthcare system; based on the categorization by Grol et al.^20^) and components (based on the taxonomy provided by the Cochrane EPOC Group).^21,22^ To assess methodologic quality, the Cochrane Risk of bias tool was applied and extended with three items specific to cluster randomized designs.^23-27^

### Analyses

To quantify and compare the effect of deimplementation strategies across studies with dichotomous and continuous outcomes, we first calculated the relative changes in use of the LVC practice for each study arm. Then, effectiveness was determined by taking the difference in relative changes between the study arms (deimplementation strategy vs. usual care or vs. another deimplementation strategy). Median scores with interquartile ranges (IQRs) were used to summarize the effectiveness of strategies across studies. This requires a reported relative change between baseline and postintervention (i.e. after applying the deimplementation strategy) or data to calculate this (i.e. volume of LVC measured preintervention and postintervention). If actual LVC (care that should not be provided according to certain appropriateness criteria) was not measured, total volume of care was used instead.

When studies compared more than one deimplementation strategy to usual care, we included the data of the most complex strategy defined by the most interventions and/or targets. When a study evaluated more than two LVC practices (e.g. various laboratory tests), we took the LVC practice with the median relative reduction. In case of two LVC practices, we selected the one with the largest relative reduction.

Differences in the effect of deimplementation strategies (i.e. relative reductions [with IQR]) were explored for several subgroups: type of outcome measured (total volume of care or actual LVC); overall risk of bias (on a study-level); type of LVC (either diagnostic, medication (i.e. antibiotics, various inappropriate medication, psychoactive medication), or nonmedication); setting (hospital care, primary care or long-term care); number of targeted groups and intervention categories; and whether the strategies were tailor-made based on preidentified influencing factors. We considered a tailor-made intervention as an intervention of which the authors of the study indicated that it was designed based on identified barriers or facilitators for deimplementation (measured by the authors themselves or identified from the literature). Furthermore, to evaluate relative effectiveness of strategies, we considered studies directly comparing deimplementation strategies. Finally, we also assessed the available data on sustainability of effects. Analyses were performed in R (version 3.6.0)^28^ and Review Manager software^29^ was used for generating the risk of bias figures. See Figure S2 and Table S3 for the risk of bias in the included studies.

## Results

### Search Results

**Figure 1.**
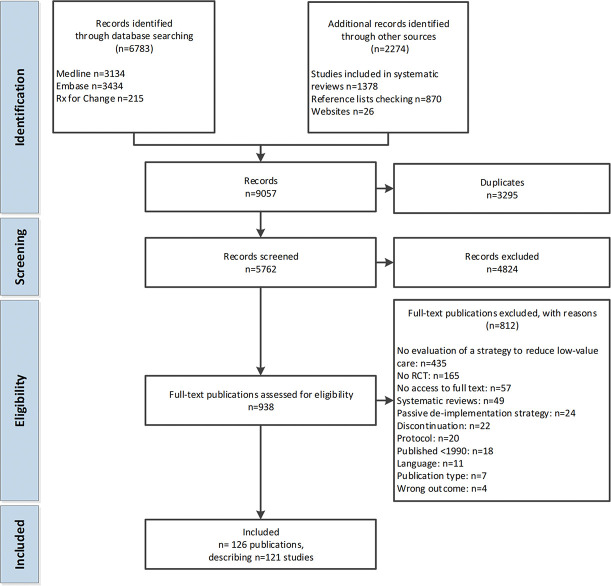
Flow chart.

The search identified 5,762 records. Based on title and abstract, 4,824 records were excluded. Full-text assessment resulted in exclusion of an additional 812 records. Main reasons for exclusion were not evaluating a deimplementation strategy (*n* = 435), or not being an RCT (*n* = 165). In total, 126 publications addressing 121 studies were included. Details of the search and selection process are presented in Figure 1 and a list of the included articles in Supplemental Appendix A (http://links.lww.com/JHQ/A189).

### Characteristics of Included Studies

One hundred six (88%) of the included studies had a cluster-randomized design with randomization on the level of healthcare centers or groups of healthcare providers (*n* = 66; 55%), healthcare providers (*n* = 33; 27%), or communities (*n* = 7; 6%). Eighty-nine (74%) were multicenter studies, 18 (15%) were community studies, and 14 (12%) took place in a single center. Strategies were compared with usual care in 109 studies (90%) and with another active deimplementation strategy in 27 studies (22%). The total adds up to more than *n* = 121, because 15 studies fell into both categories.

Most studies (*n* = 101; 83%) addressed therapeutic LVC (*n* = 89 medication; *n* = 12 nonmedication) and were performed in a primary care setting (*n* = 76; 63%) (Table 1). In 113 (93%) studies, the main goal was to reduce or not routinely provide the LVC practice, rather than to abandon it completely. Table S3, Supplemental Digital Content for the characteristics of the individual studies, http://links.lww.com/JHQ/A189

**Table 1.** Details Regarding Low-Value Care, *n* (%)

	All studies *n* = 121
Low-value care	
Diagnostic	20 (17)
Imaging	11
Laboratory tests	5
Test ordering, other	3
Breast cancer screening	1
Therapeutic, medication	89 (74)
Antibiotics	47
Inappropriate medication in general	25
Psychoactive medication	9
Other, single type of medication (i.e., anti-ulcer medication, anti-diarrhea medication, NSAIDS, pain medication)	8
Therapeutic, nonmedication	12 (10)
Blood transfusion	3
Surgery	4
Other (i.e., referrals, resource use, electronic fetal monitoring)	5
Setting	
Primary care	76 (63)
Hospital	22 (18)
Long-term care facility	13 (11)
Outpatient services	5 (4)
Other, mixed	4 (3)
Not reported	1 (1)
Definition of low value based on	
Guidelines	61 (50)
Literature (reference provided)	32 (26)
Panel	10 (8)
Other	5 (4)
Not specified	13 (11)
Aim	
Reduce/provide not routinely	113 (93)
Stop	7 (6)
Combination	1 (1)

Abbreviation: NSAID, nonsteroidal anti-inflammatory drug.

#### Deimplementation Strategies and Outcome Assessment

Deimplementation strategies were classified according to two key variables: intervention category and targeted audience (Table 2). Twenty-nine strategies (24%) addressed a single target with a single intervention. Seventeen of these strategies targeted healthcare providers, of which 11 strategies consisted of reminders (including decision support tools). In addition, 62 strategies (51%) addressed a single target as well (healthcare providers in all), but used a combination of interventions (multifaceted). About half of them (*n* = 32) combined education (meetings and/or distribution of materials) with audit and feedback (see Table S2, Supplemental Digital Content, http://links.lww.com/JHQ/A189). Another 30 multifaceted strategies (25%) were directed at multiple targets. Overall, strategies addressed a median number of 2 (IQR 2 to 3) intervention categories.

**Table 2.** Details of the Evaluated Deimplementation Strategies Regarding Interventions and Targets, *n*/*n* (%)

Intervention categories^a^ and targets	All strategies^b^ (*n* = 121)	Single target, single intervention (*n* = 29)	Single target, combination of interventions (*n* = 62)	Multiple targets^c^, combination of interventions (*n* = 30)
Targeted at provider	108/121 (89)	17/29 (59)	62/62 (100)	29/30 (97)
Educational meetings (e.g., lectures, workshops, conferences)	72/108 (67)	1/17 (6)	47/62 (76)	24/29 (83)
Distribution of educational material (e.g., publications, guidelines, pocket cards)	73/108 (68)	2/17 (12)	50/62 (81)	21/29 (72)
Reminders (including decision support tools)	31/108 (28)	11/17 (65)	15/62 (24)	5/29 (17)
Audit and feedback	58/108 (54)	3/17 (18)	40/62 (65)	15/29 (52)
Financial interventions	1/108 (1)	NA	1/62 (2)	NA
Targeted at patient	24/121 (20)	4/29 (14)	NA	20/30 (63)
Targeted at organizational context	24/121 (20)	8/29 (28)	NA	16/30 (53)
Organizational interventions (redefining roles, multidisciplinary teams, appliances, test ordering procedures and forms)	24/24 (100)	8/8 (100)	NA	16/16 (100)
Structural interventions (changing setting of care, e.g., from hospital to general practice)	1/24 (4)	NA	NA	1/16 (6)
Targeted at healthcare system	1/121 (1)	NA	NA	1/30 (3)
Regulatory interventions	NA	NA	NA	NA
Financial interventions	1/1 (100)	NA	NA	1/1 (100)

NA = not available (no studies).

a Based on taxonomy provided by the Cochrane Effective Practice and Organisation of Care (EPOC) Group.

b As a strategy can have more than one target, numbers add up to more than 121.

c Provider and patient n = 13 (43%); provider and organizational context n = 10 (33%); provider, patient and organizational context n = 5 (17%); patient and organizational context n = 1 (3%); provider, patient, and healthcare system n = 1 (3%).

All studies measured the effect on total volume of care (*n* = 79; 65%), and/or the effect on the volume of actual LVC (*n* = 47; 39%). Twelve studies (10%) evaluated sustainability of the effect.

### Effectiveness of Deimplementation Strategies

#### Deimplementation versus Usual Care (n = 109)

Of the studies comparing deimplementation with usual care, 75 (69%) reported a significant reduction compared with the usual care group.

Seventy-three of the 109 studies could be included in our quantitative analyses, because they reported the relative change from baseline for the intervention and usual care groups, or provided data to calculate this. The overall median difference in relative reductions between intervention and usual care groups in these 73 studies was 17% (IQR 7%–42%). Subsets of studies measuring actual LVC, rather than total volume (*n* = 25), and studies at overall low risk of bias (*n* = 11) showed a median difference of 46% (IQR 15%–57%) and 16% (IQR 10%–43%), respectively. Regarding type of LVC, the median difference in relative reductions was 13% (IQR 8%–24%) in studies addressing diagnostic healthcare practices, 13% (IQR 8%–22%) in studies addressing nonmedication healthcare practices, and 24% (IQR 7%–45%) in studies addressing medication (Table 3). Twenty studies aiming to reduce inappropriate medication in general achieved a median reduction of 37% (IQR 8%–51%), whereas studies targeting antibiotic use (19 studies) or psychoactive medication (6 studies) achieved median reductions of 13% (IQR 7%–43%) and 21% (IQR 12%–24%), respectively. Regarding setting, the largest median difference in relative reductions was observed for studies in long-term care facilities (43%; IQR 25%–52%), primary care (20%; IQR 8%–37%), and hospital settings (13%; IQR 5%–30%). We did not see a difference between strategies with single or multiple interventions (median difference in relative reductions of 15% [IQR 4%–33%] and 20% [IQR 9%–43%], respectively). Strategies targeting the organizational context (*n* = 5) or the provider and the patient (*n* = 9) tended to lead to a higher median difference in relative reductions; however IQRs were broad (36% [IQR 25%–52%] and 28% [IQR 28%–45%], respectively). Whether strategies were tailor-made based on preidentified barriers and facilitators (15 studies) did not result in different relative reductions (15%; IQR 10%–27%) with preidentification compared with 21% [IQR 7%–45%] without preidentification).

**Table 3.** Difference in Relative Reductions Between Deimplementation and Usual Care

	*n*	Median (IQR)
Overall	73	17% (7%–42%)
Type of LVC		
Diagnostic	14	13% (9%–24%)
Therapeutic, medication	52	24% (7%–45%)
Antibiotics	*19*	*13% (7%–43%)*
Inappropriate medication in general	*20*	*37% (8%–51%)*
Psychoactive medication	*6*	*21% (12%–24%)*
Other	*7*	*25% (11%–32%)*
Therapeutic, nonmedication	7	13% (8%–22%)
Setting		
Primary care	47	20% (8%–37%)
Hospital	13	13% (5%–30%)
Long-term care facility	9	43% (25%–52%)
Outpatient services	1	13% (NA)
Other, mixed	3	9% (9%–13%)
Deimplementation strategy		
Number of intervention categories^a^		
1	14	15% (4%–33%)
>1	59	20% (9%–43%)
Single target	37	21% (10%–46%)
Multiple targets	22	12% (7%–39%)
Strategy targets		
Provider only	45	16% (9%–37%)
Patient only	1	46% (NA)
Organizational context only	5	36% (25%–52%)
Provider and patient	9	28% (28%–45%)
Provider and organizational context	8	8% (−2%–30%)
Provider, patient, and organizational context	4	8% (7%–10%)
Provider, patient, and healthcare system	1	10% (NA)
Intervention categories		
Any education (either meetings or material, or both)	57	17% (9%–42%)
No education	16	18% (3%–38%)
Reminders	15	13% (7%–32%)
No reminders	58	18% (8%–43%)
Audit and feedback	46	20% (9%–45%)
No audit and feedback	27	14% (6%–32%)
Patient-directed intervention	15	28% (9%–43%)
No patient-directed intervention	58	17% (7%–37%)
Organizational intervention	17	9% (7%–36%)
No organizational intervention	56	20% (9%–43%)
Financial intervention	2	7% (6%–8%)
No financial intervention	71	20% (8%–43%)
Barriers and facilitators		
Preidentified	15	15% (10%–27%)
Not preidentified	58	21% (7%–45%)

NA = not available because of no or low number of studies.

a Based on taxonomy provided by the Cochrane Effective Practice and Organisation of Care (EPOC) Group (see supplementary material, http://links.lww.com/JHQ/A189).

Thirty-six studies did not report the relative change from baseline in the use of a healthcare practice or data to calculate this, but rather presented postintervention values only, (adjusted) odds ratios, hazard ratios, or mean differences (see Table S4, Supplemental Digital Content, http://links.lww.com/JHQ/A189). Half of the 24 studies targeting providers reported a significant benefit of the deimplementation strategy compared with usual care. Two of the three deimplementation strategies targeting patients and all three strategies targeting the organizational context were found to be effective compared with usual care. Of the two studies targeting providers and patients, one showed a benefit, whereas the other did not. All three studies targeting the providers and the organizational context did not find a difference of their deimplementation strategy compared with usual care.

#### Direct Comparison of Deimplementation Strategies (n = 27)

Twenty-seven studies compared a deimplementation strategy with another deimplementation strategy, of which 17 reported the relative change from baseline in the use of a low-value healthcare practice or presented data to calculate this. These studies included various (combinations of) interventions, which resulted in 28 possible direct comparisons of strategies with little overlap between studies (see Table S5, Supplemental Digital Content, http://links.lww.com/JHQ/A189). Overall, strategies with more interventions led to a larger relative reduction. Adding education or audit and feedback seemed to increase the effect of a strategy.

### Sustainability of Effect (n = 12)

Twelve studies (10%) evaluated sustainability of the effect over periods ranging from 2 to 12 months after the postintervention measurement (see Table S6, Supplemental Digital Content, http://links.lww.com/JHQ/A189). Two studies provided data on sustainability for only one of the study groups. They reported sustainable effects for the combination of educational material and audit and feedback after 9 months in one study^30^ and for the combination of educational meetings and material, audit and feedback, a patient-directed intervention, and an organizational intervention after 12 months in the other study.^31,32^ Of the remaining 10 studies, nine compared deimplementation to usual care. Four of these found a sustained effect for their strategy compared with usual care after a follow-up of 2–18 months.^33-36^ The 10th study compared two strategies targeted at providers and showed further reduction of antibiotic prescribing after 12 months for both strategies.^37^

## Limitations

Rigorous and systematic methods to explore the field of deimplementation research were applied. Despite the systematic and sensitive search strategy, it is still possible that relevant publications were missed, because of the many different terms that are used to describe the process of reducing LVC.^15^ Our focus on RCTs may be an explanation for identifying almost no disinvestment studies. Studies on the effectiveness of financial incentives at the level of individuals or organizations would have been included, though. Therefore, we believe that, overall, our set of included studies reflects the existing evidence regarding strategies to reduce LVC.

There were many different combinations of interventions used to reduce LVC. As a result, we were faced with substantial heterogeneous deimplementation strategies and were not able to disentangle the effect of a single component.

Unfortunately, it was not possible to include all studies in our quantitative analysis, because of lack of available data and heterogeneity in outcome measures (i.e., absolute numbers, proportions, ratios, rates). Using relative changes between baseline and postintervention to calculate effectiveness enabled us to include 73 of 121 studies. As a consequence of using median and interquartile ranges to summarize across studies, all studies had an equal weight in our analysis. The advantage of using the median rather than the mean is that extreme results are less likely to influence the summary estimate.^38^ Despite these challenges regarding heterogeneity and analyses, we are confident that our quantitative summary of 73 RCTs, complemented by the qualitative results, can support those who are planning to develop a deimplementation strategy.

## Discussion

Based on 121 RCTs that evaluated a strategy aimed at reducing LVC, we found that over two-thirds of the studies addressed the reduction of medication use, most frequently antibiotics. Seventy percent of study authors reported their deimplementation strategy to be successful compared with usual care and median reduction was 17% (IQR 7%–42%). Although the variation was large, the subgroup of strategies applied in long-term care facilities showed larger median differences in relative reductions than strategies in other settings. Also, strategies targeting the healthcare provider and patient or strategies targeting the organizational context seemed potentially more effective.

Apparently other factors than the number and type of strategies play a role in the effectiveness of deimplementation. Intervention-related factors such as the level of exposure to the intervention, the content of the educational materials, or the adherence to organizational interventions could have played a role, and the type of LVC, and contextual factors such as other projects that influence the deimplementation, the workplace culture, or economic/political factors.^12,39^ However, by restricting our review to RCTs, we included almost no studies addressing financial or regulatory interventions targeted at the healthcare system, because a randomized design is usually not the first choice for the evaluation of these interventions.

### Comparison with Other Studies

Our findings seem to contradict the results of Colla et al. and Cliff et al., who concluded that multicomponent interventions are potentially more effective in reducing LVC than single-component interventions.^1,17^ Our results show that single-faceted strategies have the same potential to reduce LVC practices as multifaceted strategies. A possible explanation for these differences may be that these two reviews only reported effectiveness on a dichotomous scale and included also observational studies. Colla et al. did not describe the effectiveness and the intervention types used per setting. In our study, we described the differences in the effect of deimplementation strategies (i.e., relative reductions) and explored this for several subgroups. Colla et al.^1^ reported the clinical decision support tools, education, and patient education as the most effective interventions. We found no clear differences in effect between strategies containing different components. Rietbergen et al. showed that half of the deimplementation studies were effective, but because of heterogeneity and a lack of studies, no conclusions could be drawn on which strategy is most effective. Also, this study did not quantify the effects and only 55% of the studies had a control group.^16^ It has been suggested that for changing behavior, it is not the number or type of interventions that matters, but the fact that an implementation strategy is context-specific and addresses existing influencing factors.^2,40-42^ We identified relatively few studies that reported how assessment of barriers and facilitators informed the design of their strategy. However, this does not automatically mean that they had not been taken into account when designing the deimplementation strategy. Future studies should therefore describe in more detail which barriers and facilitators have been identified and how these were translated into the subsequent strategies.

## Conclusions

Most active deimplementation strategies identified were successful in reducing LVC, achieving a median relative reduction of 17%. These results should encourage healthcare professionals and policymakers to initiate their own deimplementation projects. We found no signs that a specific type or number of interventions works best for deimplementation. Future deimplementation studies should therefore map the relevant contextual factors and tailor their interventions. These contextual factors may include other projects that influence the deimplementation, individual health professional factors (e.g., awareness, knowledge, beliefs, routines), the social context (e.g., culture of the network or leadership), or economic/political factors (e.g., financial arrangements). In addition, insights into sustainability of effects will further advance our understanding regarding the optimal approach to reduce LVC.

## Implications

We found that a considerable reduction of LVC was possible. However, it will depend on the baseline level of LVC and the context whether the actual impact of a strategy is clinically meaningful. A reduction of 17% may be insignificant for one LVC practice, yet it could mean a substantial improvement of quality of care in other practices (e.g., when serious adverse events are prevented). It is essential that future deimplementation studies provide all essential information needed to interpret and apply their results. This includes knowledge on contextual factors, intervention details, and on sustainability of effect. Authors should use the relevant guidelines aimed at transparant reporting, such as the Standards for Reporting Implementation Studies (StaRI) Statement, the Standards for Quality Improvement Reporting Excellence (SQUIRE 2.0), and the Template for Intervention Description and Replication (TIDieR) checklist.^43-45^

## Authors' Biographies

**Pauline Heus, PhD**, is an assistant professor at Cochrane Netherlands, Julius Center for Health Sciences and Primary Care, University Medical Center Utrecht, Utrecht University Str. 6.131, P.O. Box 85500, 3508 GA Utrecht, The Netherlands. Her main research areas of interest are the translation of evidence into clinical practice (implementation and deimplementation) and aspects of responsible research.

**Simone van Dulmen, PhD,** is a senior researcher, Radboud University Medical Center, Radboud Institute for Health Sciences, IQ healthcare, PO Box 9101, 6500 HB, Nijmegen, The Netherlands. Her main research activities are related to deimplement low-value care and implementation of high-value care, and research related to sustainable healthcare.

**Jan-Willem Weenink, PhD,** is an assistant Professor, Erasmus School of Health Policy & Management (ESHPM), Burg. Oudlaan 50, P.O. Box 1738, 3000 DR Rotterdam, The Netherlands. His research is related to health services research.

**Christiana A. Naaktgeboren, PhD,** is an epidemiologist with expertise regarding methodology of diagnostic research. At the time of the research presented in this manuscript, she was an assistant professor at the Julius Center for Health Sciences and Primary Care, University Medical Center Utrecht, Utrecht University Str. 6.131, P.O. Box 85500, 3508 GA Utrecht, the Netherlands.

**Toshihiko Takada, MD, PhD,** is a general practitioner and associate professor at the department of general medicine of Shirakawa Satellite for Teaching And Research (STAR), Fukushima Medical University, 2-1, Toyochi Kamiyajiro, Shirakawa, Fukushima, 961-0005, Japan. At the time of the research presented in this manuscript, he worked as an assistant professor at the Julius Center for Health Sciences and Primary Care, University Medical Center Utrecht, Utrecht University Str. 6.131, P.O. Box 85500, 3508 GA Utrecht, the Netherlands.

**Eva W. Verkerk, PhD,** is a senior researcher, Radboud University Medical Center, Radboud Institute for Health Sciences, IQ healthcare, PO Box 9101, 6500 HB, Nijmegen, the Netherlands. Her main research areas of interest are the translation of evidence into clinical practice (implementation and deimplementation).

**Isabelle Kamm, BSc,** is a medical student affiliated to Cochrane Netherlands, Julius Center for Health Sciences and Primary Care, University Medical Center Utrecht, Utrecht University Str. 6.131, P.O. Box 85500, 3508 GA Utrecht, the Netherlands. She contributes to various research projects and also to the Netherlands Trial Register.

**Maarten van der Laan, MD, PhD,** is a vascular surgeon and Head of the Vascular Surgery Training Program at the University Medical Center Groningen, Department Surgery, PO Box 30.001, 9700 RB, Groningen, the Netherlands. He is also the vice chairman of the Consortium Quality of Care of the NFU (Dutch Federation of University Medical Centers).

**Lotty Hooft, PhD,** is the director of Cochrane Netherlands, Professor Evidence Synthesis and Knowledge Translation and Head of the Epidemiology department of the Julius Center for Health Sciences and Primary Care, University Medical Center Utrecht, Utrecht University Str. 6.131, P.O. Box 85500, 3508 GA Utrecht, the Netherlands. Areas of her interest and expertise are methodology of various types of evidence syntheses (i.e. reviews on diagnostic test accuracy and prognostic research), clinical trial registration, responsible research practices, and knowledge translation.

**Rudolf B. Kool, PhD,** is a senior researcher, Radboud University Medical Center, Radboud Institute for Health Sciences, IQ healthcare, PO Box 9101, 6500 HB, Nijmegen, the Netherlands. His main research activities are related to deimplement low-value care and implementation of high-value care, and e-health.

## Supplementary Material

SUPPLEMENTARY MATERIAL
