# Supplementary material for: What are Effective Strategies to Reduce Low-Value Care? An Analysis of 121 Randomized Deimplementation Studies
Source: J Healthc Qual. 2023 Jun 29;45(5):261–71. doi: 10.1097/JHQ.0000000000000392 (PMC10461725; doi:10.1097/JHQ.0000000000000392)
Supplement: SUPPLEMENTARY MATERIAL [file jhq-45-261-s003.docx]

# Supplementary file A

To manuscript: *What are effective strategies to reduce low-value care? An analysis of 121 randomized de-implementation studies.*

## Systematic review – details on methods and results

### Methods

#### Data Sources and Searches

An information specialist searched EMBASE, MEDLINE, and Rx for Change databases on November 11^th^, 2019. Search terms included synonyms for de-implementation and low-value care. In addition, we selected systematic reviews by the Cochrane Effective Practice and Organisation of Care (EPOC) group from the Cochrane Library that were potentially relevant based on title and abstract and checked all included primary studies for eligibility for our review. Similarly, we checked all primary studies included in systematic reviews identified by our search. Furthermore, websites of healthcare quality improvement organizations were searched, and reference lists of the articles we screened on full text were used as an additional source of potentially relevant studies. Details regarding the search are available in Appendix 1.

#### Study Selection

Randomized controlled trials (RCTs) in which a strategy aimed at reducing low-value care was studied, that were published after 1990 in English, German, French, or Dutch, were eligible. For relevant protocols of RCTs identified by the search it was checked whether the study had been published as a full text.
Low-value care was defined as a healthcare practice that according to the study authors was considered inappropriate. We included studies assessing the effect of a strategy on the incidence of low-value care, e.g. new prescriptions or diagnostic test orders. Studies evaluating the cessation of long-term medication use (discontinuation in the context of an individual patient’s care) were excluded. Studies on guideline adherence were only included when it was possible to extract information on reduction of healthcare practices.
Pairs of authors independently screened titles and abstracts, and subsequently full texts of potentially eligible publications (CN, EV, JWW, PH, RK, SvD, and TT). Discrepancies were resolved through discussion and, when necessary, a third author was consulted.

#### Data Extraction and critical appraisal

Data of eligible publications were extracted by one author and checked by a second author (CN, EV, IK, JWW, LH, MvdL, PH, RK, SvD, and TT). To ensure consistency between the reviewers, we developed and piloted a standardized electronic data extraction form that included study characteristics (low-value care being de-implemented, targets and components of the de-implementation strategy, study design) and effect measures (outcomes). We distinguished four different target levels of a de-implementation strategy (hereafter just called targets): provider, patient, organizational context (including social context) and healthcare system, based on the categorization by Grol et al.^1^ The components used in de-implementation strategies were classified into nine categories based on the taxonomy provided by the Cochrane EPOC Group (see Appendix 2).^2,3^
Two authors independently assessed risk of bias by applying the Cochrane Risk of bias tool (CN, EV, IK, PH, or TT).^4^ In addition to the seven domains of this tool, three issues specific to cluster randomized designs were assessed: recruitment bias, unit of analysis error, and concern regarding baseline imbalances.^5-8^ Studies were classified as overall at low risk of bias when they had 1) an adequate random sequence generation, 2) scored a low risk of bias for all three domains related to cluster randomized designs, and 3) no high risk of bias due to unconcealed allocation, detection bias, attrition bias, or reporting bias, with unclear risk of bias for a maximum of two of these domains.

### Results

#### Risk of bias

Within most bias domains, the majority of studies were judged to be at a low risk of bias (see Figure S2, table S3). However, as interventions often could not be blinded, a high risk of performance bias was considered for 104 (86%) of the studies, potentially leading to contamination of control arms (and thereby dilution of intervention effects). Details on randomization procedure were not provided in 51 (42%) (sequence generation) and 85 (70%) (allocation concealment) randomized controlled trials, leading to an unclear risk of selection bias in these trials. Risk of reporting bias was judged to be unclear in 99 (82%) trials, as study protocols were not available, and high in 7 (6%) trials. Risk of detection bias was judged to be low in half of the studies. Risk of attrition bias was judged to be low in the majority of the studies (75 trials; 62%). With regard to the risk of bias domains addressing a clustered design (relevant for n=106 included studies), low risk of bias was found in the majority of the studies (n=66 [62%] for recruitment bias, n=82 [77%] for unit of analysis error, and n=78 [74%] for baseline imbalances). In 18 (15%) studies the overall risk of bias was judged to be low.

## Supplementary tables

### Description of Included studies

Table S1 Included studies (n=121)

| Reference | Country | Unit of randomization | Type of low-value care | Low-value care | Setting | Strategy target | Strategy – intervention categories* |
| --- | --- | --- | --- | --- | --- | --- | --- |
| Allard 2001^9^ | Canada | Individual participant | Medication | Medication general | Primary care | Provider | Educational material, audit and feedback |
| Althabe 2004^10^ | Argentina, Brazil, Cuba, Guatemala, Mexico | Healthcare center / practice / group of providers | Non-medication | Surgery | Hospital | Provider and organization | Educational material, organizational intervention (policy of mandatory second opinion) |
| Althabe 2008^11^ | Argentina, Uruguay | Healthcare center / practice / group of providers | Non-medication | Surgery | Hospital | Provider | Educational meetings, educational material and audit and feedback |
| Altiner 2007^12^ | Germany | Provider | Medication | Antibiotics | Primary care | Provider and patient | Educational meetings and patient directed interventions (leaflets and posters) |
| Anderson 1996^13^ | Canada (British Colombia) | Provider | Medication | Medication general | Primary care | Provider | Educational meetings and reminders |
| Awad 2006^14^ | Sudan | Healthcare center / practice / group of providers | Medication | Antibiotics | Primary care | Provider | Educational meetings, audit and feedback |
| Baker 2003^15^ | UK | Healthcare center / practice / group of providers | Diagnostic | Pathology tests | Primary care | Provider | Educational material, audit and feedback |
| Bates 1999^16^ | USA | Individual participant | Diagnostic | Laboratory tests | Hospital | Provider | Reminders |
| Bearcroft 1994^17^ | UK | Healthcare center / practice / group of providers | Diagnostic | Imaging | Primary care | Provider | Educational material |
| Berings 1994 ^18^ | Belgium | Provider | Medication | Benzodiazepines | Primary care | Provider | Educational meetings and educational material |
| Bhatia 2014^19^ | USA | Provider | Diagnostic | Imaging | Hospital | Provider | Educational meetings, educational material, audit and feedback |
| Bhatia 2017^20^ | Canada, USA | Provider | Diagnostic | Imaging | Hospital | Provider | Educational meetings, educational material, audit and feedback |
| Boersma 2019^21^ | The Netherlands | Provider | Medication | Medication general | Hospital | Provider | Reminders |
| Bourgeois 2010^22^ | USA | Healthcare center / practice / group of providers | Medication | Antibiotics | Outpatient services | Provider | Reminders |
| Braybrook 1996^23^ | United Kingdom | Healthcare center / practice / group of providers | Medication | Antibiotics | Primary care | Provider | Educational meetings, educational material and audit and feedback |
| Braybrook 2000^24^ | United Kingdom | Healthcare center / practice / group of providers | Medication | NSAID | Primary care | Provider | Educational meetings, educational material and audit and feedback |
| Bregnhøj 2009^25^ | Denmark | Healthcare center / practice / group of providers | Medication | Medication general | Primary care | Provider | Educational meetings, audit and feedback |
| Briel 2006^26^ | Switserland | Provider | Medication | Antibiotics | Primary care | Provider | Educational meetings, educational material |
| Bryant 2011^27^ | New Zealand | Healthcare center / practice / group of providers | Medication | Medication general | Primary care | Organization | Organizational interventions (comprehensive pharmaceutical care, discussion between pharmacist and patient’s general practitioner) |
| Campins 2017^28^ | Spain | Patient | Medication | Medication general | Primary care | Organization | Organizational interventions (pharmacist medication review and recommendations) |
| Cánovas 2009^29^ | Spain | Healthcare center / practice / group of providers | Medication | Medication general | Primary care | Provider | Educational meetings, educational material, audit and feedback |
| Carney 2012^30^ | USA | Provider | Diagnostic | Screening | Other (mammo-graphy registries) | Provider | Educational material, audit and feedback |
| Christakis 2001^31^ | USA | Provider | Medication | Antibiotics | Other (outpatient clinics and hospitals) | Provider | Reminders |
| Clyne 2018^32,33^ | Ireland | Healthcare center / practice / group of providers | Medication | General | Primary care | Provider and patient | Educational meetings, educational material, patient directed interventions (educational material) |
| Coenen 2004^34^ | Belgium | Provider | Medication | Antibiotics | Primary care | Provider and patient | Educational meetings, educational material, reminders, patient directed interventions (educational material as part of public campaign also including television spots, and radio messages) |
| Crotty 2004^35^ | Australia | Healthcare center / practice / group of providers | Medication | Medication general | Long term care facility | Provider and organization | Educational meetings, organizational interventions (multidisciplinary case conferences) |
| Culbertson 1990^36^ | USA | Community | Medication | Anti-ulcer medication | Unknown | Provider | Educational meetings, educational material |
| Daley 2018^37^ | Canada | Individual participant | Medication | Antibiotics | Hospital | Organization | Organizational interventions (modified report) |
| Davies 2002^38^ | Canada | Healthcare center / practice / group of providers | Non-medication | Electronic fetal monitoring | Hospital | Provider | Educational meetings, educational material, audit and feedback |
| De Burgh 1995^39^ | Australia | Provider | Medication | Benzodiazepines | Primary care | Provider | Educational meetings, educational material |
| Dey 2004^40^ | UK | Healthcare center / practice / group of providers | Diagnostic | Imaging | Primary care | Provider and organization | Educational meetings, educational material, organizational interventions (access to fast-track physiotherapy and provision of triage service) |
| Dolovich 1999^41^ | Canada | Community | Medication | Antibiotics | Primary care | Provider and organization | Educational material, organizational interventions (manufacturer detailing) |
| Doyne 2004^42^ | USA | Healthcare center / practice / group of providers | Medication | Antibiotics | Outpatient services | Provider, patient, and organization | Educational meetings, educational material, audit and feedback, patient directed intervention (educational material) and organizational interventions (opinion leaders) |
| Dranitsaris 2001^43^ | Canada, USA | Individual participant | Medication | Antibiotics | Hospital | Provider and organization | Reminders, organizational interventions (involvement of pharmacist for medication review) |
| Eccles 2001^44^ | UK | Healthcare center / practice / group of providers | Diagnostic | Imaging, referral | Primary care | Provider | Educational material, reminders, audit and feedback |
| Eltayeb 2005^45^ | Sudan | Healthcare center / practice / group of providers | Medication | Medication general | Primary care | Provider | Educational meetings, educational material, audit and feedback |
| Engers 2005^46^ | The Netherlands | Provider | Non-medication | Referrals (in analyses; pain medication | Primary care | Provider | Educational meetings, educational material |
| Fender 1999^47^ | United Kingdom | Healthcare center / practice / group of providers | Non-medication | Referrals | Primary care | Provider | Educational meetings, educational material |
| Fenton 2016^48,49^ | USA | Provider | Diagnostic | Imaging | Primary care | Provider | Educational meetings |
| Field 2009^50^ | Canada | Healthcare center / practice / group of providers | Medication | Medication renal insufficiency | Long term care facility | Provider | Reminders |
| Fine 2003^51^ | USA | Provider | Medication | Antibiotics | Hospital | Provider | Educational material, reminders |
| Finkelstein 2001^52^ | USA | Healthcare center / practice / group of providers | Medication | Antibiotics | Primary care | Provider, patient and organization | Educational meetings, educational material, patient directed interventions (educational materials via e-mail and in waiting rooms), organizational interventions (peer leader) |
| Finkelstein 2008^53^ | USA | Community | Medication | Antibiotics | Primary care | Provider and patient | Educational meetings, educational material, audit and feedback, patient directed interventions (educational material via letter, newsletters, posters, handouts website; training) |
| Flottorp 2002^54^ | Norway | Healthcare center / practice / group of providers | Diagnostic | Laboratory tests (in analyses); Antibiotics | Primary care | Provider, patient and system | Educational meetings, educational material, reminders, patient directed interventions (educational material in electronic and paper format), financial interventions (increase in fee for telephone consultations) |
| Fossey 2006^55^ | UK | Healthcare center / practice / group of providers | Medication | Medication general | Long term care facility | Provider | Educational meetings, audit and feedback |
| Francis 2009^56^ | UK | Healthcare center / practice / group of providers | Medication | Antibiotics | Primary care | Provider | Educational meetings, educational material |
| Frankenthal 2014^57^ | Israel | Individual participant | Medication | Medication general | Long term care facility | Organization | Organizational interventions (involvement of pharmacist for medication review) |
| French 2013^58^ | Australia | Healthcare center / practice / group of providers | Diagnostic | Imaging | Primary care | Provider | Educational meetings, educational material |
| Gallagher 2011^59^ | Ireland | Individual participant | Medication | Medication general | Hospital | Provider | Reminders, audit and feedback |
| Garcia-Gollarte 2014^60^ | Spain | Provider | Medication | Medication general | Long term care facility | Provider | Educational meetings, educational material, reminders, audit and feedback |
| Gjelstad 2013^61^ | Norway | Healthcare center / practice / group of providers | Medication | Antibiotics | Primary care | Provider and organization | Educational meetings, reminders, audit and feedback, organizational interventions (peer academic detailer; software tool for registration) |
| Goldberg 2001^62^ | USA | Community | Non-medication | Surgery | Other (primary care and hospitals) | Provider, patient, and organization | Educational meetings, educational material, audit and feedback, patient directed interventions (brochures and office wall charts), organizational interventions (opinion leaders) |
| Hadiyono 1996^63^ | Indonesia | Healthcare center / practice / group of providers | Non-medication | Injections | Primary care | Provider and patient | Educational meetings, patient directed interventions (interactional group  discussion with providers and patients) |
| Hemminki 2008^64^ | Finland | Healthcare center / practice / group of providers | Non-medication | Surgery | Outpatient services | Provider and patient | Educational meetings, educational material, patient directed interventions (information leaflet) |
| Hodnett 1996^65^ | Canada | Healthcare center / practice / group of providers | Medication | Epidural analgesia | Hospital | Provider | Educational meetings, educational material, audit and feedback |
| Holm 1990^66^ | Denmark | Healthcare center / practice / group of providers | Medication | Sedatives | Primary care | Provider | Educational meetings, educational material, audit and feedback |
| Ilett 2000^67^ | Australia | Provider | Medication | Antibiotics | Primary care | Provider | Educational meetings, educational material |
| Kerry 2000^68^ | UK | Healthcare center / practice / group of providers | Diagnostic | Imaging | Primary care | Provider | Educational material, audit and feedback |
| Kirkham 2019^69^ | Canada | Healthcare center / practice / group of providers | Medication | Antipsychotics | Long term care facility | Provider | Educational meetings, educational material |
| Lagerløv 2000^70^ | Norway | Provider | Medication | Antibiotics | Primary care | Provider | Educational meetings, audit and feedback |
| Lemiengre 2018^71^ | Belgium | Healthcare center / practice / group of providers | Medication | Antibiotics | Primary care | Patient and organization | Patient directed interventions (eliciting parental concern and providing a safety net, information leaflet) and organizational (reducing clinicians’ uncertainty with an objective inflammatory parameter) interventions |
| Linder 2009^72^ | USA | Healthcare center / practice / group of providers | Medication | Antibiotics | Primary care | Provider | Educational material, reminders |
| Loeb 2005^73^ | Canada | Healthcare center / practice / group of providers | Medication | Antibiotics | Long term care facility | Provider | Educational meetings, educational material, reminders |
| Lundborg 1999^74^ | Sweden | Provider | Medication | Antibiotics | Primary care | Provider | Educational meetings and audit and feedback |
| Macfarlane 2002^75^ | United Kingdom | Individual participant | Medication | Antibiotics | Primary care | Patient | Patient directed interventions (information leaflet) |
| Mainous III 2000^76^ | USA | Provider | Medication | Antibiotics | Primary care | Provider and patient | Audit and feedback, patient directed interventions (educational material) |
| Martens 2007^77^ | Netherlands | Healthcare center / practice / group of providers | Medication | Antibiotics | Primary care | Provider | Reminders |
| McIsaac 2002^78^ | Canada | Provider | Medication | Antibiotics | Primary care | Provider | Educational material, reminders |
| Meador 1997^79^ | USA | Healthcare center / practice / group of providers | Medication | Antipsychotics | Long term care facility | Provider | Educational meetings, educational material |
| Meeker 2014^80^ | USA | Provider | Medication | Antibiotics | Primary care | Patient | Patient directed interventions (poster-sized letters in examination rooms) |
| Meeker 2016^81^ | USA | Healthcare center / practice / group of providers | Medication | Antibiotics | Primary care | Provider | Educational material, reminders, audit and feedback |
| Metlay 2007^82^ | USA | Community | Medication | Antibiotics | Hospital | Provider, patient and organization | Educational meetings, educational material, audit and feedback, patient directed interventions (educational material in waiting and examination rooms; computerized education in waiting room) , organizational interventions (clinical leaders) |
| Milos 2013^83^ | Sweden | Individual participant | Medication | Medication general | Other (primary care and long term care facility) | Provider | Audit and feedback |
| Monette 2007^84^ | Canada | Healthcare center / practice / group of providers | Medication | Antibiotics | Long term care facility | Provider | Educational material, audit and feedback |
| Nilsson 2002^85^ | Sweden | Provider | Medication | Calcium channel blockers, anti-peptic ulcer/dyspepsia | Primary care | Provider | Educational meetings, educational material, audit and feedback, and organizational interventions (local opinion leaders) |
| Oakeshott 1994^86^ | UK | Healthcare center / practice / group of providers | Diagnostic | Imaging | Primary care | Provider | Educational material |
| Pagaiya 2005^87^ | Thailand | Healthcare center / practice / group of providers | Medication | Medication general | Primary care | Provider | Educational meetings, educational material, audit and feedback |
| Patterson 2010^88^ | Northern-Ireland | Healthcare center / practice / group of providers | Medication | Psychoactive medication | Long term care facility | Provider | Educational meetings, reminders, audit and feedback |
| Payne 1991^89^ | USA | Healthcare center / practice / group of providers | Non-medication | Resources | Hospital | Provider | Audit and feedback |
| Persell 2016^90^ | USA | Provider | Medication | Antibiotics | Primary care | Provider | Educational material, reminders, audit and feedback |
| Pettersson 2011^91^ | Sweden | Healthcare center / practice / group of providers | Medication | Antibiotics | Long term care facility | Provider | Educational meetings, educational material, audit and feedback |
| Pimlott 2003^92^ | Canada | Provider | Medication | Benzodiazepines | Primary care | Provider | Educational material, audit and feedback |
| Pit 2007^93^ | Australia | Healthcare center / practice / group of providers | Medication | Medication general | Primary care | Provider | Educational meetings, educational material, audit and feedback, financial intervention |
| Pitkälä 2014^94^ | Finland | Healthcare center / practice / group of providers | Medication | Medication general | Long term care facility | Provider | Educational meetings, educational material |
| Pshetizky 2003^95^ | Israel | Individual participant | Medication | Antibiotics | Primary care | Patient | Patient directed interventions (short, structured, verbal explanation) |
| Raebel 2007^96^ | USA | Individual participant | Medication | Medication general | Outpatient services | Organization | Organizational interventions (medication review by pharmacist) |
| Regev 2011^97^ | Israel | Provider | Medication | Antibiotics | Outpatient services | Provider | Educational meetings, educational material, audit and feedback |
| Rognstad 2013^98^ | Norway | Healthcare center / practice / group of providers | Medication | Medication general | Primary care | Provider | Educational meetings, audit and feedback |
| Rothschild 2007^99^ | USA | Provider | Non-medication | Transfusions | Hospital | Provider | Educational meetings, educational material, reminders |
| Rubin 2001^100^ | Australia | Healthcare center / practice / group of providers | Non-medication | Transfusions | Hospital | Provider and organization | Educational meetings, educational material, audit and feedback |
| Samore 2005^101^ | USA | Community | Medication | Antibiotics | Primary care | Provider | Educational meetings, educational material, reminders |
| Santoso 1996^102^ | Indonesia | Community | Medication | Antimicrobials, anti-diarrhea | Primary care | Provider | Educational meetings, educational material |
| Schectman 2003^103^ | USA | Healthcare center / practice / group of providers | Diagnostic | Imaging | Primary care | Provider and patient | Educational meetings, educational material, audit and feedback, patient directed interventions (written and videotaped educational information) |
| Schmidt 1998^104^ | Sweden | Healthcare center / practice / group of providers | Medication | Psychotropic drugs | Long term care facility | Organization | Organizational interventions (multidisciplinary team meetings) |
| Seager 2005^105^ | UK | Healthcare center / practice / group of providers | Medication | Antibiotics | Primary care | Provider and patient | Educational meetings, educational material, patient directed interventions (information leaflet) |
| Shojania 1998^106^ | USA | Provider | Medication | Antibiotics, vancomycin | Hospital | Provider | Reminders |
| Simon 2006^107^ | USA | Healthcare center / practice / group of providers | Medication | Medication general | Primary care | Provider | Educational meetings, reminders |
| Solomon 2001^108^ | USA | Healthcare center / practice / group of providers | Medication | Antibiotics | Hospital | Provider | Educational meetings, audit and feedback |
| Søndergaard 2003^109^ | Denmark | Provider | Medication | Antibiotics | Primary care | Provider | Educational material, audit and feedback |
| Soumerai 1998^110^ | USA | Healthcare center / practice / group of providers | Medication | Prophylactic lidocaine | Hospital | Provider and organization | Educational meetings, educational material, audit and feedback, organizational interventions (opinion leaders), structural interventions (revision of clinical pathways) |
| Spinewine 2007^111^ | Belgium | Individual participant | Medication | Medication general | Hospital | Organization | Organizational interventions (pharmacist involvement) |
| Tamblyn 2003^112^ | Canada | Provider | Medication | Medication general | Primary care | Provider | Reminders |
| Taylor 2005^113^ | USA | Individual participant | Medication | Antibiotics | Primary care | Patient | Patient directed interventions (educational material [video with accompanying pamphlet]) |
| Terrel 2009^114^ | USA | Provider | Medication | Medication general | Hospital | Provider | Reminders |
| Thomas 2006^115^ | Scotland (United Kingdom) | Healthcare center / practice / group of providers | Diagnostic | Laboratory tests | Primary care | Provider | Educational material, reminders, audit and feedback |
| Tierney 1990^116^ | USA | Provider | Diagnostic | Test ordering | Primary care | Provider | Reminders |
| Trietsch 2017^117^ | Netherlands | Healthcare center / practice / group of providers | Medication | Laboratory tests; antibiotics (in analyses) | Primary care | Provider | Educational meetings, audit and feedback |
| Urbiztondo 2017^118^ | Argentina, Bolivia, Uruguay, and Paraguay | Healthcare center / practice / group of providers | Medication | Antibiotics | Primary care | Provider and patient | Educational meetings, educational material, audit and feedback, patient directed interventions (educational material) |
| Van Driel 2007^119^ | Belgium | Healthcare center / practice / group of providers | Medication | Antibiotics | Primary care | Provider | Educational meetings, educational material |
| Van Eijk 2001^120^ | Netherlands | Healthcare center / practice / group of providers | Medication | Antidepressants | Primary care | Provider | Educational meetings, educational material, audit and feedback |
| Van Wijk^121^ | Netherlands | Healthcare center / practice / group of providers | Diagnostic | Laboratory tests | Primary care | Provider | Reminders |
| Verstappen 2003^122,123^ | Netherlands | Healthcare center / practice / group of providers | Diagnostic | Test ordering | Primary care | Provider | Educational meetings, educational material, audit and feedback |
| Vierhout 1995^124^ | Netherlands | Individual participant | Non-medication | Referrals | Primary care | Organization | Organizational interventions (joint consultation sessions of surgeons and general practitioners) |
| Voorn 2017^125^ | Netherlands | Healthcare center / practice / group of providers | Non-medication | Blood transfusion | Hospital | Provider | Educational meetings, educational material, audit and feedback |
| Weber 2008^126^ | USA | Healthcare center / practice / group of providers | Medication | Medication general | Primary care | Provider and organization | Reminders, organizational interventions (involvement of pharmacist or geriatrician) |
| Wei 2017^127,128^ | China | Healthcare center / practice / group of providers | Medication | Antibiotics | Primary care | Provider and patient | Educational meetings, educational material, audit and feedback, patient directed interventions (information leaflet and video) |
| Weller 2003^129^ | Australia | Healthcare center / practice / group of providers | Diagnostic | PSA test | Primary care | Provider and patient | Educational meetings, educational material, audit and feedback, patient directed interventions (educational material) |
| Welschen 2004^130^ | Netherlands | Healthcare center / practice / group of providers | Medication | Antibiotics | Primary care | Provider and patient | Educational meetings, audit and feedback, patient directed interventions (educational material) |
| Wilson 2003^131,132^ | Australia | Provider | Medication | Antibiotics | Primary care | Provider, patient and organization | Educational meetings, educational material, audit and feedback, patient directed interventions (information sheets and posters), organizational interventions (prescription pads) |
| Winkens 1995^133^ | Netherlands | Patient | Diagnostic | Laboratory tests; physiology; imaging (in analysis) | Primary care | Provider | Audit and feedback |
| Zwar 1999^134^ | Australia | Provider | Medication | Antibiotics | Primary care | Provider and organization | Educational meetings, educational material, audit and feedback, organizational interventions (consensus panel) |

RCT: randomized controlled trial
* Based on taxonomy provided by the Cochrane Effective Practice and Organization of Care (EPOC) Group^2,3^

### De-implementation strategies

Table S2 De-implementation strategies in included studies

| Single target, single intervention  (n=29) | n | Single target, combination of  interventions  (n=62) | n | Multiple targets, combination of interventions  (n=30) | n |
| --- | --- | --- | --- | --- | --- |
| Provider | **17** | **Provider** | **62** | **Provider and patient** | **13** |
| Reminders | 11 | Education (meetings & material) + audit and feedback | 17 | Education (meetings & material) + audit and feedback + patient directed intervention | 5 |
| Audit and feedback | 3 | Education (meetings & material) | 14 | Education (meetings & material) + patient directed intervention | 3 |
| Educational material | 2 | Educational meetings + audit and feedback | 8 | Educational meetings + patient directed intervention | 2 |
| Educational meetings | 1 | Educational material + audit and feedback | 7 | Education (meetings & material) + reminders + patient directed intervention | 1 |
| Patient | **4** | Educational material + reminders + audit and feedback | 4 | Educational meetings + audit and feedback + patient directed intervention | 1 |
| Organizational context | **8** | Education (meetings & material) + reminders | 3 | Audit and feedback + patient directed intervention | 1 |
|  |  | Educational material + reminders | 3 | **Provider and organizational context** | **10** |
|  |  | Educational meetings + reminders | 2 | Education (meetings & material) + audit and feedback + organizational intervention | 2 |
|  |  | Educational meetings + reminders + audit and feedback | 1 | Educational material + organizational intervention | 2 |
|  |  | Education (meetings & material) + reminders + audit and feedback | 1 | Reminders + organizational intervention | 2 |
|  |  | Reminders + audit and feedback | 1 | Educational meetings + organizational intervention | 1 |
|  |  | Education (meetings & material) + audit and feedback + financial intervention | 1 | Education (meetings & material) + organizational intervention | 1 |
|  |  |  |  | Educational meetings + reminders + audit and feedback + organization | 1 |
|  |  |  |  | Education (meetings & material) + audit and feedback + organizational intervention + structural intervention | 1 |
|  |  |  |  | **Provider, patient, and organizational context** | **5** |
|  |  |  |  | Education (meetings & material) + audit and feedback + patient directed + organizational intervention | 4 |
|  |  |  |  | Education (meetings & material) + patient directed + organizational intervention | 1 |
|  |  |  |  | **Patient and organizational context** | **1** |
|  |  |  |  | **Provider, patient, and system** | **1** |
|  |  |  |  | Education (meetings & material) + reminders + patient directed intervention + financial intervention | 1 |

### Risk of bias assessment


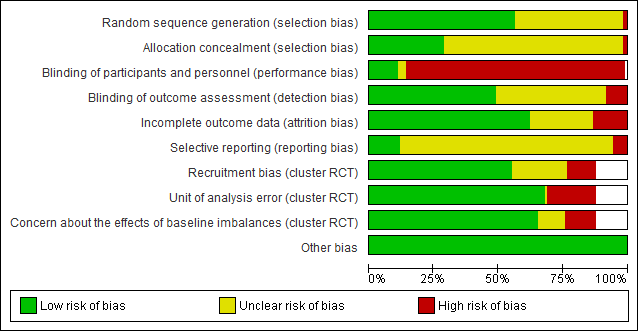


Figure S2 Risk of bias graph

Table S3 Risk of bias in included studies

| Study | Random sequence generation (selection bias) | Allocation concealment (selection bias) | Blinding of participants and personnel (performance bias) | Blinding of outcome assessment (detection bias) | Incomplete outcome data (attrition bias) | Selective reporting (reporting bias) | Recruitment bias  (*cluster RCT*) | Unit of analysis error (*cluster RCT*) | Concern about the effects of baseline imbalances (*cluster RCT*) | Other bias | Low overall risk of bias* |
| --- | --- | --- | --- | --- | --- | --- | --- | --- | --- | --- | --- |
| Allard 2001^9^ | Unclear | Unclear | High | Low | Low | Unclear | NA | NA | NA | None | No |
| Althabe 2004^10^ | Low | Low | High | Unclear | Low | Unclear | Low | Low | Low | None | Yes |
| Althabe 2008^11^ | Unclear | Unclear | High | Low | Low | Low | Unclear | Low | Low | None | No |
| Altiner 2007^12^ | Low | Unclear | High | Unclear | Low | Unclear | High | Low | Low | None | No |
| Anderson 1996^13^ | Low | Unclear | High | Unclear | Low | Unclear | Low | Low | Low | None | No |
| Awad 2006^14^ | Unclear | Unclear | High | Unclear | Low | Unclear | Low | Low | Unclear | None | No |
| Baker 2003^15^ | Low | Unclear | High | Unclear | Low | Unclear | Low | Low | Unclear | None | No |
| Bates 1999^16^ | Unclear | Low | High | Low | Low | Unclear | NA | NA | NA | None | No |
| Bearcroft 2004^17^ | Low | Unclear | Low | Low | Unclear | Unclear | Unclear | High | Unclear | None | No |
| Berings 1994^18^ | Low | Unclear | High | Unclear | Low | Unclear | Low | High | Low | None | No |
| Bhatia 2014^19^ | Low | Unclear | High | Low | Low | Unclear | Low | Low | Low | None | Yes |
| Bhatia 2017^20^ | Unclear | Low | High | Low | Low | Unclear | Low | Low | Low | None | No |
| Boersma 2019^21^ | Low | Unclear | High | Unclear | Low | Low | High | Low | High | None | No |
| Bourgeois 2010^22^ | Unclear | Unclear | High | Unclear | Unclear | High | Unclear | Low | High | None | No |
| Braybrook 1996^23^ | Low | Unclear | High | Unclear | High | Unclear | Unclear | High | Low | None | No |
| Braybrook 2000^24^ | Unclear | Unclear | High | Unclear | High | Unclear | Unclear | High | Low | None | No |
| Bregnhøj 2009^25^ | Low | Unclear | High | Low | High | Unclear | High | Low | High | None | No |
| Briel 2006^26^ | Unclear | Unclear | High | Low | Low | Unclear | Low | Low | Low | None | No |
| Bryant 2011^27^ | Low | Unclear | High | Low | High | Unclear | High | High | High | None | No |
| Campins 2017^28^ | Low | Low | High | Low | Low | Unclear | NA | NA | NA | None | Yes |
| Cánovas 2009^29^ | Low | Unclear | Low | Unclear | High | Unclear | High | Low | High | None | No |
| Carney 2012^30^ | Unclear | Unclear | High | Unclear | Unclear | Unclear | Low | Low | Low | None | No |
| Christakis 2001^31^ | Low | Unclear | High | Unclear | Unclear | Unclear | High | Low | Unclear | None | No |
| Clyne 2016^32,33^ | Low | Low | High | Low | Low | Low | Low | Low | Low | None | Yes |
| Coenen 2004^34^ | Low | Low | High | High | Low | Low | Unclear | Low | Low | None | No |
| Crotty 2004^35^ | Low | Low | High | Low | Low | Unclear | High | Low | Low | None | No |
| Culbertson 1999^36^ | High | Unclear | High | Unclear | Low | Unclear | Unclear | High | High | None | No |
| Daley 2018^37^ | Low | Low | High | High | Low | High | NA | NA | NA | None | No |
| Davies 2002^38^ | Low | Low | High | Low | High | Unclear | Low | Low | Unclear | None | No |
| De Burgh 1995^39^ | Unclear | Unclear | High | High | Low | Unclear | Unclear | Low | Low | None | No |
| Dey 2004^40^ | Low | Unclear | High | High | Low | Unclear | High | Low | Low | None | No |
| Dolovich 1999^41^ | Low | Unclear | High | Low | Low | Unclear | Unclear | Low | Low | None | No |
| Doyne 2004^42^ | Low | Unclear | High | Unclear | Low | Unclear | Low | High | High | None | No |
| Dranitsaris 2001^43^ | Low | Unclear | High | High | Low | Unclear | NA | NA | NA | None | No |
| Eccles 2001^44^ | Low | Unclear | High | Low | Low | Unclear | Low | Low | High | None | No |
| Eltayeb 2005^45^ | Unclear | Unclear | High | Low | Low | Unclear | Unclear | High | Low | None | No |
| Engers 2005^46^ | Low | Low | High | Unclear | Low | Unclear | High | Low | Low | None | No |
| Fender 1999^47^ | Low | Unclear | High | Unclear | Unclear | Unclear | High | Low | Unclear | None | No |
| Fenton 2016^48,49^ | Unclear | Unclear | High | Low | Low | Low | Low | Low | Low | None | No |
| Field 2009^50^ | Unclear | Unclear | High | Unclear | Unclear | Unclear | Low | Low | Low | None | No |
| Fine 2003^51^ | Unclear | Unclear | High | Low | Low | Unclear | Unclear | Low | Low | None | No |
| Finkelstein 2001^52^ | Unclear | Unclear | High | Low | Unclear | Unclear | Low | Low | Low | None | No |
| Finkelstein 2008^53^ | Low | Unclear | High | Low | Low | Unclear | Low | Low | Low | None | Yes |
| Flottorp 2002^54^ | Low | Unclear | Low | Low | High | Unclear | Low | Low | Low | None | No |
| Fossey 2006^55^ | Low | Unclear | High | Low | Unclear | Unclear | Low | Low | Low | None | No |
| Francis 2009^56^ | Low | Low | High | Low | Low | Low | Low | Low | Low | None | Yes |
| Frankenthal 2014^57^ | Low | Low | High | Low | Low | Unclear | NA | NA | NA | None | Yes |
| French 2013^58^ | Low | Low | High | Low | Low | High | Low | Low | High | None | No |
| Gallagher 2011^59^ | Low | Low | High | Low | Low | Low | NA | NA | NA | None | No |
| Garcia-Gollarte 2014^60^ | Low | Unclear | High | Low | Low | Unclear | Low | High | Low | None | No |
| Gjelstad 2013^61^ | Unclear | Low | Low | Low | Low | Unclear | Low | Low | Low | None | No |
| Goldberg 2001^62^ | Low | Low | Unclear | Low | Low | Unclear | High | Low | Low | None | No |
| Hadiyono 1996^63^ | Unclear | Unclear | High | Low | Low | Unclear | Unclear | High | Unclear | None | No |
| Hemminki 2008^64^ | Low | Unclear | High | Unclear | Low | Unclear | Low | High | Low | None | No |
| Hodnett 1996^65^ | Low | Low | High | Unclear | Unclear | Unclear | Low | Low | High | None | No |
| Holm 1990^66^ | Low | Low | High | Unclear | Low | Unclear | Low | Low | Low | None | Yes |
| Ilett 2000 ^67^ | Unclear | Unclear | High | Unclear | Unclear | Unclear | Low | Low | Low | None | No |
| Kerry 2000^68^ | Unclear | Unclear | High | Unclear | Low | Unclear | Unclear | High | Unclear | None | No |
| Kirkham 2019^69^ | Low | Unclear | High | Unclear | Low | Unclear | Low | Low | Low | None | No |
| Lagerløv 2000^70^ | Unclear | Unclear | Low | Unclear | Low | Unclear | Unclear | High | Low | None | No |
| Lemiengre 2018^71^ | Unclear | Unclear | High | Unclear | Unclear | Unclear | Unclear | Low | Low | None | No |
| Linder 2009^72^ | Unclear | Unclear | High | Low | Low | Unclear | Low | Low | Low | None | No |
| Loeb 2005^73^ | Low | Low | High | Low | Unclear | Low | Low | Low | Low | None | Yes |
| Lundborg 1999^74^ | Low | Unclear | High | Unclear | Unclear | Unclear | Low | Low | Low | None | No |
| Macfarlane 2002^75^ | Unclear | Unclear | High | Low | Low | Unclear | NA | NA | NA | None | No |
| Mainous III 2000^76^ | Unclear | Unclear | High | Unclear | Unclear | Unclear | Low | Low | Unclear | None | No |
| Martens 2007^77^ | Unclear | Unclear | Low | Unclear | Low | Unclear | Unclear | High | High | None | No |
| McIsaac 2002^78^ | Unclear | Unclear | High | Unclear | High | Unclear | Unclear | High | Unclear | None | No |
| Meador 1997^79^ | Unclear | Unclear | High | Low | Unclear | Unclear | Low | High | Low | None | No |
| Meeker 2014^80^ | Low | Unclear | High | Low | Low | Low | Low | Unclear | Low | None | No |
| Meeker 2016^81^ | Low | Unclear | High | Unclear risk of bias | Low | Low | Low | Low | Low | None | Yes |
| Metlay 2007^82^ | Low | Unclear | High | Unclear | Low | Unclear | Low | Low | Low | None | No |
| Milos 2013^83^ | Low | Low | High | Unclear | Low | Unclear | NA | NA | NA | None | Yes |
| Monette 2007^84^ | Low | Unclear | High | High | Low | Unclear | Low | Low | Low | None | No |
| Nilsson 2002^85^ | Unclear | Unclear | High | Unclear | Unclear | Unclear | Low | Low | Low | None | No |
| Oakeshott 1994^86^ | Unclear | Unclear | High | Unclear | Low | Unclear | High | Low | High | None | No |
| Pagaiya 2005^87^ | Unclear | Unclear | High | Unclear | Unclear | Unclear | Unclear | Low | Low | None | No |
| Patterson 2010^88^ | Low | Low | High | Low | Unclear | Unclear | Unclear | Low | Low | None | No |
| Payne 1991^89^ | Unclear | Unclear | High | Unclear | High | Unclear | Low | Low | Unclear | None | No |
| Persell 2016^90^ | Low | Low | High | Low | Low | Low | Low | High | Low | None | No |
| Pettersson 2011^91^ | Low | High | High | High | High | Unclear | Unclear | Low | Low | None | No |
| Pimlott 2003^92^ | Unclear | Unclear | Low | Low | Low | Unclear | Low | Low | Low | None | No |
| Pit 2007^93^ | Low | Low | High | Low | Unclear | Unclear | High | High | Low | None | No |
| Pitkälä 2014^94^ | Low | Low | High | Unclear | High | Unclear | Low | High | High | None | No |
| Pshetizky 2003^95^ | Unclear | Low | High | Unclear | Low | Unclear | NA | NA | NA | None | No |
| Raebel 2007^96^ | Low | Unclear | Low | Low | Unclear | Unclear | NA | NA | NA | None | No |
| Regev 2011^97^ | Unclear | Unclear | High | Unclear | Low | High | Low | Low | Low | None | No |
| Rognstad 2013^98^ | Unclear | Low | Unclear | Low | Low | Unclear | Low | Low | Low | None | No |
| Rothschild 2007^99^ | Low | Unclear | Low | Low | Low | Unclear | Low | Low | Unclear | None | No |
| Rubin 2001^100^ | Low | Unclear | High | Low | High | Unclear | Unclear | High | High | None | No |
| Samore 2005^101^ | Unclear | Unclear | High | Low | Low | Unclear | Unclear | Low | Low | None | No |
| Santoso 1996^102^ | Unclear | Unclear | High | Unclear | Unclear | Unclear | Unclear | High | High | None | No |
| Schectman 2003^103^ | Unclear | Low | High | Low | Unclear | Unclear | Low | Low | Low | None | No |
| Schmidt 1998^104^ | Unclear | Unclear | High | Unclear | High | Unclear | Low | High | Low | None | No |
| Seager 2005^105^ | Low | Unclear | High | Unclear | High | Unclear | Unclear | Low | Low | None | No |
| Shojania 1998^106^ | High | Unclear | High | Unclear | Unclear | Unclear | Low | Low | Low | None | No |
| Simon 2006^107^ | Unclear | Unclear | High | Low | Low | Unclear | Low | Low | Low | None | No |
| Solomon 2001^108^ | Unclear | Unclear | High | Low | Unclear | Unclear | Unclear | Low | Low | None | No |
| Søndergaard 2003^109^ | Unclear | Unclear | High | Unclear | Unclear | Unclear | Low | Low | Low | None | No |
| Soumerai 1998^110^ | Unclear | Unclear | Low | Low | Unclear | Unclear | Low | Low | Low | None | No |
| Spinewine 2007^111^ | High | High | High | Low | Low | Unclear | NA | NA | NA | None | No |
| Tamblyn 2003^112^ | Unclear | Unclear | High | Low | Unclear | Unclear | Low | Low | Low | None | No |
| Taylor 2005^113^ | Low | Unclear | High | Unclear | Low | Unclear | NA | NA | NA | None | No |
| Terrell 2009^114^ | Low | Unclear | High | Low | Low | High | Low | High | Low | None | No |
| Thomas 2006^115^ | Low | Low | High | Low | Low | High | Low | Low | Low | None | No |
| Tierney 1990^116^ | Unclear | Unclear | High | Low | High | Unclear | Low | Low | Unclear | None | No |
| Trietsch 2017^117^ | Low | Low | Unclear | Low | Low | Low | Low | Low | Low | None | Yes |
| Urbiztondo 2017^118^ | Low | Low | High | High | Low | Unclear | Unclear | Low | Low | None | No |
| Van Driel 2007^119^ | Unclear | Unclear | High | High | Unclear | High | High | Low | Low | None | No |
| Van Eijk 2001^120^ | Unclear | Unclear | High | Unclear | Low | Unclear | Low | Low | Low | None | No |
| Van Wijk 2001^121^ | Low | Low | Unclear | Unclear | Low | Unclear | Low | Low | Low | None | Yes |
| Verstappen 2003^122,123^ | Low | Low | Low | Low | Low | Unclear | Low | Low | Low | None | Yes |
| Vierhout 1995^124^ | Unclear | Unclear | High | High | High | Unclear | NA | NA | NA | None | No |
| Voorn 2017^125^ | Low | Low | High | Unclear | Low | Low | Low | Low | Low | None | Yes |
| Weber 2008^126^ | Unclear | Unclear | Low | Low | Low | Unclear | Low | Low | Low | None | No |
| Wei 2017^127,128^ | Low | Unclear | High | Low | Low | Low | Low | Low | Low | None | Yes |
| Weller 2003^129^ | Low | Low | High | Low | Low | Unclear | Low | Low | Low | None | Yes |
| Welschen 2004^130^ | Low | Unclear | High | Low | Low | Unclear | Low | Low | Low | None | Yes |
| Wilson 2003^131,132^ | Low | Unclear | High | Unclear | Unclear | Unclear | Low | Low | Low | None | No |
| Winkens 1995^133^ | Unclear | Unclear | Low | Unclear | Unclear | Unclear | Unclear | Unclear | Unclear | None | No |
| Zwar 1999^134^ | Unclear | Unclear | High | Unclear | Low | Unclear | Low | Low | Low | None | No |

NA: not applicable
*Studies were classified as low risk of bias when they had 1) an adequate random sequence generation, 2) scored a low risk of bias for all three domains related to cluster randomised designs and 3) no high risk of bias due to unconcealed allocation, detection bias, attrition bias, or reporting bias, with unclear risk of bias for a maximum of two of these domains.

### Effectiveness

Table S4 Results for studies not providing (data to calculate) the relative change from baseline in the use of a healthcare practice (n=36)

| Study | Type of low-value care | Targeted at | Number of Intervention categories* | Intervention categories* | Outcome description | Results for intervention vs. control group, as reported by study authors |
| --- | --- | --- | --- | --- | --- | --- |
| Fenton 2016^48,49^ | Diagnostic | Provider | 1 | Educational meetings | Test ordering in intervention relative to control group | Adjusted OR 1.07 (95%CI 0.49 to 2.32) |
| Bearcroft 1994^17^ | Diagnostic | Provider | 1 | Educational material | Inappropriate test requests for chest radiography | 5.7% vs. 8.2%; p=0.016 |
| Bourgeois 2010^22^ | Therapeutic | Provider | 1 | Reminders | Proportion of visits with a viral diagnosis that generated an antibiotic prescription | 17.9% vs. 15.7%; p=0.129 |
| Christakis 2001^31^ | Therapeutic | Provider | 1 | Reminders | Proportion of patients with otitis media who were treated without antibiotics | Change of -4.3% vs. -16.8%; p=0.095 (“indicating that the intervention may have had some protective effect against a trend of generally increasing antibiotic use.”) |
| Martens 2007^77^ | Medication | Provider | 1 | Reminders | Number of antibiotic prescriptions per 1000 patients per general practitioner | 28.2 (95% CI 20.8 to 44.5) vs. 39.7 (95% CI 29.7 to 64.1); p=0.2 |
| Field 2009^50^ | Therapeutic | Provider | 1 | Reminders | Inappropriate drug orders | RR 0.78 (95% CI 0.64 to 0.95) |
| Shojania 1998^106^ | Therapeutic | Provider | 1 | Reminders | Initiated or renewed order for vancomycin | 7.4 versus 10.3 orders per physician; p=0.02 |
| Terell 2009^114^ | Therapeutic | Provider | 1 | Reminders | Proportion of potentially inappropriate medication | OR= 0.59 (95% CI 0.41 to 0.85); absolute reduction of 2.0% (95% CI 0.7 to 3.3%) |
| Engers 2005^46^ | Non-medication | Provider | 2 | Education (meetings & material) | Referrals | OR 0.8 (95% CI 0.5 to 1.4) |
| Fender 1999^47^ | Therapeutic | Provider | 2 | Education (meetings & material) | Referrals | OR 0.64; 95% (95% CI 0.41 to 0.99) |
| Van Driel 2007^119^ | Therapeutic | Provider | 2 | Education (meetings & material) | Prescribing first choice antibiotic | Adjusted OR 1.07 (95% CI 0.34 to 3.37) |
| Fine 2003^51^ | Therapeutic | Provider | 2 | Educational material + reminders | Conversion to oral antibiotics | HR 1.23, (95%CI 1.00 to 1.52) |
| Francis 2009^56^ | Therapeutic | Provider | 2 | Education (meetings & material) | Primary care reconsultation within first two weeks | OR 0.75 (95% CI 0.41 to 1.38) |
| French 2013^58^ | Diagnostic | Provider | 2 | Education (meetings & material) | X-ray referrals | Incident rate ratio 0.83 (95% CI 0.61 to 1.12) |
| Kirkham 2019^69^ | Therapeutic | Provider | 2 | Education (meetings & material) | Inappropriate antipsychotic prescribing rates | Weighted mean change from baseline to 12-month follow-up: 4.6% (SD 2.8%), p<0.0001, representing a 16.1% (SD 17.0%) relative reduction.  Adjusted OR 0.73 (95% CI 0.48 to 0.94)  (Stepped wedge design with control group as pre-intervention and intervention group as post-intervention.) |
| Pitkälä 2014^94^ | Therapeutic | Provider | 2 | Education (meetings & material) | Harmful medication prescriptions | Change of -11.7 vs. 3.4; p=0.022 (adjusted for age, sex, and comorbidities) |
| Linder 2009^72^ | Therapeutic | Provider | 2 | Educational material + reminders | Antibiotic prescribing rate | Adjusted OR 0.8 (95%CI 0.6 to 1.2) |
| McIsaac 2002^78^ | Therapeutic | Provider | 2 | Educational material + reminders | Unnecessary antibiotic prescriptions | Adjusted OR 0.76 (95% CI 0.42 to 1.40) |
| Lagerløv 2000^70^ | Medication | Provider | 2 | Educational meetings + audit and feedback | Proportion of inappropriate treatments | Intervention group relative to control group: change of -9.7%; p=0.0004 |
| Solomon 2001^108^ | Therapeutic | Provider | 2 | Educational meetings + audit and feedback | Days of unnecessary target antibiotic use per 2-week interval | Mean 5.5 vs. 8.8; Risk reduction for a day of unnecessary target antibiotic: 41% (95% CI 44% to 78%) |
| Bhatia 2014^19^ | Diagnostic | Provider | 3 | Education (meetings & material) + audit and feedback | Ordering an appropriate test | OR 2.7 (95% CI 1.5 to 5.1) |
| Bhatia 2017^20^ | Diagnostic | Provider | 3 | Education (meetings & material) + audit and feedback | Ordering a rarely appropriate test | OR 0.75 (95% CI 0.57 to 0.99) |
| Holm 1990^66^ | Therapeutic | Provider | 3 | Education (meetings & material) + audit and feedback | Prescription rate | Change of -6.8% vs. -16.8%; NS |
| Loeb 2005^73^ | Therapeutic | Provider | 3 | Education (meetings & material) + reminders | Courses of antimicrobials for suspected urinary tract infections per 1000 resident days | Weighted mean difference − 0.49 (95%CI − 0.93 to − 0.06) |
| Macfarlane 2002^75^ | Therapeutic | Patient | 1 | Patient directed intervention | Antibiotics taken | RR 0.76 (95%CI 0.59 to 0.97) |
| Pshetizky 2003^95^ | Therapeutic | Patient | 1 | Patient directed intervention | Antibiotics administered | 37% versus 63%; p<0.0001 |
| Taylor 2005^113^ | Therapeutic | Patient | 1 | Patient directed intervention | Mean number of visits per patient where antibiotics were prescribed for otitis media | 1.7 (SD 2.1) vs. 1.9 (SD 2.4); p=0.23 |
| Daley 2018^37^ | Therapeutic | Organizational context | 1 | Interventions directed at organizational context | Rate of treatment of asymptomatic bacteriuria | 10/35 (37.1%) vs. 24/41 (58.5%); p=0.016 |
| Raebel 2007^96^ | Therapeutic | Organizational context | 1 | Intervention directed at organizational context | Medication dispensed | 2.9% vs. 5.5%; p<0.001 |
| Vierhout 1995^124^ | Therapeutic | Organizational context | 1 | Intervention directed at organizational context | Referrals | 35% vs. 68%; p<0.01 |
| Hemminki 2008^64^ | Therapeutic | Provider and patient | 3 | Education (meetings & material) + patient directed intervention | C-sections | Adjusted OR 1.29 (95%CI 0.99 to 1.68) |
| Seager 2005^105^ | Therapeutic | Provider and patient | 3 | Education (meetings & material) + patient directed intervention | Inappropriate antibiotic prescriptions | OR 0.33 (95% 0.21 to 0.54) |
| Dolovich 1999^41^ | Therapeutic | Provider and organizational context | 2 | Educational material + intervention directed at organizational context | Change in mean percentage market share for third-line agents (cefixime, amoxicillin clavulanate, or cefaclor) between preintervention and postintervention time periods | 0.06% vs. -0.07%; p=0.85 |
| Dranitsaris 2001^43^ | Therapeutic | Provider and organizational context | 2 | Reminders + intervention directed at organizational context | Proportion of cefotaxime orders that were inconsistent with hospital guidelines | 25% vs. 31%; p=0.24 |
| Lemiengre 2018^71^ | Therapeutic | Patient and organizational context | 2 | Interventions directed at patient and at organizational context | Antibiotic prescribing | Crude OR 1.07 (95% CI 0.61 to 1.88); adjusted OR 1.21 (95% CI 0.66 to 2.22) |
| *Only addressing sustainability, no results directly post intervention* | | | | | | |
| Clyne 2016^32,33^ | Therapeutic | Provider and patient | 3 | Education (meetings & material) + patient directed intervention | New instances of potentially inappropriate prescribing | At 1 year post intervention  16 (in 12 [13 %] participants vs. 18 (in 18 [20 % participants]; p=0.38 |

CI: confidence interval; HR: hazard ratio; NS: not statistically significant; OR: odds ratio; RR: relative risk; SD: standard deviation;
* Based on taxonomy provided by the Cochrane Effective Practice and Organization of Care (EPOC) Group^2,3^

Table S5 Difference between relative reductions in use of a healthcare practice for direct comparison of de-implementation strategies (28 comparisons; n=17 studies)

| Study | Type of low-value care | Number of intervention categories compared | Intervention categories compared (interventions vs. control intervention) | Difference in relative reductions from baseline  for intervention vs. control groups* |
| --- | --- | --- | --- | --- |
| Targeted at provider only | | | | |
| Eccles 2001^44^ | Diagnostic | 1 vs. 1 | Audit and feedback vs. reminders | -12.1% |
| Thomas 2006^115^ | Diagnostic | 2 vs. 1 | Educational material + audit and feedback vs. reminders | 11.6% |
| Cánovas 2009^29^ |  | 2 vs. 1 | Educational meetings + educational material vs. reminders | 62.2% |
| Simon 2006^107^ | Therapeutic | 2 vs. 1 | **Educational meetings + reminders vs. reminders** | 4.8% |
| Anderson 1996^13^ | Therapeutic |  |  | 8.8% |
| Awad 2006^14^ | Therapeutic | 2 vs. 1 | **Educational meetings (academic detailing)** + audit and feedback vs. Audit and feedback | 43.4% |
| Awad 2006^14^ | Therapeutic | 2 vs. 1 | **Educational meetings (seminar)** + audit and feedback vs. Audit and feedback | 32.1% |
| Eltayeb 2005^45^ | Therapeutic | 3 vs. 1 | **Educational meetings (acad. detailing) + educational material** + audit and feedback vs. Audit and feedback | 34.3% |
| Eltayeb 2005^45^ | Therapeutic | 3 vs. 1 | **Educational meetings (seminars) + educational material** + audit and feedback vs. Audit and feedback | 27.2% |
| Rubin 2001^100^ | Therapeutic | 3 vs. 2 | **Educational meetings** + educational material + audit and feedback vs. Educational material + audit and feedback | -11.8% |
| Weller 2003^129^ | Diagnostic | 4 vs. 3 | **Educational meetings** + educational material + audit and feedback + organizational intervention vs. Educational material + audit and feedback + organizational intervention | 24.0% |
| Bregnhøj 2009^25^ | Therapeutic | 2 vs. 1 | Educational meetings + **audit and feedback** vs. Educational meetings | 56.2% |
| Eccles 2001^44^ | Diagnostic | 2 vs. 1 | Reminders + **audit and feedback** vs. reminders | 7.3% |
| Meeker 2016^81^ | Therapeutic | 2 vs. 1 | Educational material + **audit and feedback** vs. educational material | 35.8% |
| Eccles 2001^44^ | Diagnostic | 2 vs. 1 | **Reminders** + audit and feedback vs. audit and feedback | 19.4% |
| Meeker 2016^81^ | Therapeutic | 2 vs. 1 | Educational material + **reminders (accountable justification)** vs. educational material | 31.9% |
| Meeker 2016^81^ | Therapeutic | 2 vs. 1 | Educational material + **reminders (suggested alternatives)** vs. educational material | 26.8% |
| Thomas 2006^115^ | Diagnostic | 3 vs. 2 | Educational material + **reminders** + audit and feedback vs. educational material + audit and feedback | -1.8% |
| Thomas 2006^115^ | Diagnostic | 3 vs. 1 | **Educational material** + reminders + **audit and feedback** vs. reminders | 9.8% |
| Santoso 1996^102^ | Therapeutic | 2 vs. 2 | Educational meetings **(small meeting**) + educational material vs. Educational meetings **(seminar)** + educational material | -5.9% |
| Awad 2006^14^ | Therapeutic | 2 vs. 2 | Educational meetings (**academic detailing**) + audit and feedback vs. Educational meetings **(seminar)** + audit and feedback | 11.3% |
| Eltayeb 2005^45^ | Therapeutic | 3 vs. 3 | Educational meetings **(acad. detailing**) + educational material + audit and feedback vs. Educational meetings **(seminars**) + educational material + audit and feedback | 7.1% |
| Van Eijk 2001^120^ | Therapeutic | 3 vs. 3 | Educational meetings **(individual visit**) + educational material + audit and feedback vs. Educational meetings (**group visit**) + educational material + audit and feedback | -29.8% |
| Targeted at either providers or patients | | | | |
| Mainous III 2000^76^ | Therapeutic | 1 vs. 1 | Audit and feedback vs. patient directed interventions | -14.0% |
| Targeted both providers and patients | | | | |
| Mainous III 2000^76^ | Therapeutic | 2 vs. 1 | **Audit and feedback** + patient directed interventions vs. patient directed interventions | -5.0% |
| Urbiztondo 2017^118^ | Therapeutic | 4 vs. 3 | Educational meetings + Educational material + **audit and feedback** + patient directed interventions vs. Educational meetings + Educational material + patient directed interventions | 6.9% |
| Mainous III 2000^76^ | Therapeutic | 2 vs. 1 | Audit and feedback + **patient directed interventions** vs. audit and feedback | 9.0% |
| Samore 2005^101^ | Therapeutic | 4 vs. 1 | **Educational meetings + educational material + reminders** + patient directed interventions vs. patient directed interventions | 10.5% |
| Targeted both provider and organizational context | | | | |
| Weller 2003^129^ | Diagnostic | 4 vs. 3 | **Educational meetings + educational material + audit and feedback + organizational intervention vs. Educational material + audit and feedback + organizational intervention** | 24.0% |
| Targeted provider, patient, and organizational context | | | | |
| Doyne 2004^42^ | Therapeutic | 5 vs. 2 | **Educational meetings + educational material + audit and feedback + patient directed intervention + organizational intervention** vs. educational material + audit and feedback | 4% |

*A negative difference in relative reductions means a smaller effect (i.e. smaller reduction) for the intervention compared to the control intervention.

Table S6 Results for studies reporting on sustainability of the effect (n=12)

| Reference | Type of low-value care | Strategy target | Strategy – intervention categories* | Outcome description | Baseline | Post intervention | End of study | Duration of follow-up (after post intervention measurement) |
| --- | --- | --- | --- | --- | --- | --- | --- | --- |
| Althabe 2008^11^ | Non-medication | Provider | Educational meetings, educational material, audit & feedback | Episotomy rate | 41.1% | 29.9% | 28.1% | 18 months |
|  |  | - | No intervention(s) |  | 43.5% | 44.5% | 45.1% |  |
| Altiner 2007^12^ | Medication | Provider, patient | Educational meetings, patient directed intervention | Prescription rate | 36.4% | 29.4% | 36.7% | 10.5 months |
|  |  | - | No intervention(s) |  | 54.7% | 59.4% | 65.8% |  |
| Awad 2006^14^ | Medication | Provider | Educational meetings (academic detailing), audit & feedback | Inappropriate antibiotic encounters | 24.7% | 6.0% | 3.0% | 2 months |
|  |  | Provider | Audit & feedback |  | 21.7% | 14.7% | 13.0% |  |
|  |  | Provider | Educational meetings (seminar), audit & feedback |  | 24.3% | 8.7% | 5.3% |  |
|  |  | - | No intervention(s) |  | 25.0% | 24.0% | 23.0% |  |
| Braybrook 1996^23^ | Medication | Provider | Educational meetings, educational material, audit & feedback | Changes in prescribing patterns | NR | -5% | -8% | 12 months |
|  |  | Provider | Educational material, audit & feedback |  | NR | -2 | -6% |  |
| Bryant 2011^27^ | Medication | Organizational context | Organizational intervention | Mean number of inappropriate medication per patient | 2.5 | 1.6 | 1.4 | 6 months |
|  |  | - | No intervention(s) |  | 2.1 | 2.1 | 1.4 |  |
| Carney 2012^30^ | Diagnostic | Provider | Educational material, audit & feedback | Mammography recall | 11.2% | 10.8% | 10.4% | 9 months |
|  |  | - | No intervention(s) |  | 8.7% | 9.2% | NR |  |
| Clyne 2016^32,33^ | Medication | Provider, patient | Educational meetings, educational material, patient directed intervention | No. of patients who have a new instance of potentially inappropriate medication | NR | NR | 13% | 12 months |
|  |  | - | No intervention(s) |  | NR | NR | 20% |  |
| Gallagher 2011^59^ | Medication | Provider | Reminders, audit & feedback | Medication appropriateness index (MAI) score | 10 | 3 | 3 | 6 months |
|  |  | - | No intervention(s) |  | 8 | 8 | 8 |  |
| Monette 2007^84^ | Medication | Provider | Educational material, audit & feedback | Nonadherent prescriptions | 43.8% | 28.4% | “…nonadherent antibiotic prescriptions remained lower in the experimental group, but this did not reach statistical significance (OR 50.48, 95% CI 50.23–1.02).” | 3 months |
|  |  | - | No intervention(s) |  |  |  |  |  |
| Tierney 1990^116^ | Diagnostic | Provider | Reminders | Number of tests ordered | 1.81 | 1.56 | 7.7% lower than in control group | 19 weeks |
|  |  | - | No intervention(s) |  | 1.72 | 1.82 | NR |  |
| Weller 2003^129^ | Diagnostic | Provider & Organization | Educational meetings, educational material, audit & feedback, organizational intervention | PSA tests per 100 consultations | 1.6 | 1.32 | 1.6 | 6 months |
|  |  | Provider & Organization | Educational material, audit & feedback, organizational intervention |  | 1.85 | 1.97 | 2.1 |  |
|  |  | - | No intervention(s) |  | 1.73 | 2.2 | 1.9 |  |
| Wilson 2003^131,132^ | Medication | Provider, Patient & Organization | Educational meetings, educational material, audit & feedback, patient directed intervention, organizational intervention | Prescriptions per 100 Medicare services | 7.52 | 6.9 | 6.74 | 12 months |
|  |  | Provider & Patient | Educational meetings, educational material, audit & feedback, patient directed intervention |  | 7.16 | 7.12 | NR |  |

NR: not reported

* Based on taxonomy provided by the Cochrane Effective Practice and Organization of Care (EPOC) Group^2,3^

## References

## Appendix 1 Search strategy

### Databases

Date: November 11, 2019

**MEDLINE (Ovid)**

(((((obsole* or (("not" or "no longer") adj (effective or essential or efficient)) or ineffective or uneffective or low-value or overuse* or inappropriate or "old habits") adj4 ("health system" or healthcare or care or policy or policies or practice or technology or procedure* or treatment* or intervention* or "services*" or strateg* or "clinical use" or referral* or diagnosis or regulatory or approach or prescrib* or therap*)) or (overtest* or overdiagnos*)) adj5 (reduce or avoid or minimise or discontinu* or minimise or decreas* or stop or stopping or revers* or replace* or avert or "trim down" or (cut adj (down or back)) or substitute or decrement)) or ("de-implementation" or deimplementation or "do-not-do" or "deadopt*" or decommission*)).mp.

**Embase**

#1 (((((obsole* or (("not" or "no longer") adj (effective or essential or efficient)) or ineffective or uneffective or low-value or overuse* or inappropriate or "old habits") adj4 ("health system" or healthcare or care or policy or policies or practice or technology or procedure* or treatment* or intervention* or "service*" or strateg* or "clinical use" or referral* or diagnosis or regulatory or approach or prescrib* or therap*)) or (overtest* or overdiagnos*)) adj5 (reduce or avoid or minimise or discontinu* or minimise or decreas* or stop or stopping or revers* or replace* or avert or "trim down" or (cut adj (down or back)) or substitute or decrement)) or ("de-implementation" or deimplementation or "do-not-do" or "deadopt*" or decommission*)).mp.

#2 limit 1 to (conference abstract or conference paper or conference review)

#3 1 not 2

**Rx for change**

"de-adoption" OR "Decrease use" OR "abandoning" OR "Discontinue" OR "discontinuation" OR "Abandon" OR "Reassess" OR "reassessment" OR "Obsolete" OR "Medical reversal" OR "Re-invest" OR "Withdraw*" OR "de-implementation" OR "Reduc*" OR "Decline in use" OR "Health technology reassessment" OR "Change in use" OR "De-implement*" OR "De-list" OR "De-commission" OR "Do not do" OR "Reallocation" OR "relinquishing" OR "Over use" OR "Stop" OR "Inappropriate use" OR "Relinquish*" OR "Ineffective" OR "Misuse" OR "Re-appraisal" OR "Re-prioritization" OR "Clinical redesign" OR "Disadoption" OR "Redeploy" OR "Reversal" OR "Drop in use" OR "stopping"

### Systematic reviews

*Either identified by the database searches (see above) or selected from the set systematic reviews by the Cochrane Effective Practice and Organization of Care (EPOC) Group in the Cochrane Database of Systematic Reviews, Issue 10, 2019*

1. Akbari A, Mayhew A, Al-Alawi MA, Grimshaw J, Winkens R, et al. Interventions to improve outpatient referrals from primary care to secondary care. Cochrane Database of Systematic Reviews. 2008:CD005471.
2. Akl EA, Kairouz VF, Sackett KM, Erdley WS, Mustafa RA, et al. Educational games for health professionals. Cochrane Database of Systematic Reviews. 2013(3).
3. Alber JM, Brewer NT, Melvin C, Yackle A, Smith JS, et al. Reducing overuse of cervical cancer screening: A systematic review. Preventive Medicine. 2018;116:51-59.
4. Alldred DP, Kennedy MC, Hughes C, Chen TF, Miller P. Interventions to optimise prescribing for older people in care homes. Cochrane Database of Systematic Reviews. 2016(2).
5. Andrews T, Thompson M, Buckley DI, Heneghan C, Deyo R, et al. Interventions to influence consulting and antibiotic use for acute respiratory tract infections in children: A systematic review and Meta-Analysis. PLoS ONE. 2012;7.
6. Arditi C, Rège‐Walther M, Durieux P, Burnand B. Computer‐generated reminders delivered on paper to healthcare professionals: effects on professional practice and healthcare outcomes. Cochrane Database of Systematic Reviews. 2017(7).
7. Arnold SR, Straus SE. Interventions to improve antibiotic prescribing practices in ambulatory care. Cochrane Database of Systematic Reviews. 2005(4).
8. Axt-Adam P, van der W, J C, van der D. Influencing behavior of physicians ordering laboratory tests: a literature study. Medical Care. 1993;31(9):784-794.
9. Baker R, Camosso‐Stefinovic J, Gillies C, Shaw EJ, Cheater F, et al. Tailored interventions to address determinants of practice. Cochrane Database of Systematic Reviews. 2015(4).
10. Bell S, McLachlan AJ, Aslani P, Whitehead P, Chen TF. Community pharmacy services to optimise the use of medications for mental illness: A systematic review. Australia and New Zealand Health Policy. 2005;2.
11. Boonacker CWB, Hoes AW, Dikhoff MJ, Schilder AGM, Rovers MM. Interventions in health care professionals to improve treatment in children with upper respiratory tract infections. International Journal of Pediatric Otorhinolaryngology. 2010;74:1113-1121.
12. CADTH. Proton pump inhibitor cessation programs: a review of the clinical effectiveness, cost-effectiveness, and guidelines. Ottawa: Canadian Agency for Drugs and Technologies in Health; 2014/02// 2014.
13. Castelino RL, Bajorek BV, Chen TF. Targeting suboptimal prescribing in the elderly: a review of the impact of pharmacy services. The Annals of Pharmacotherapy. 2009;43:1096-1106.
14. Chambers J, Salem M, Neumann PJ. Disinvesting in low-value care: Opportunities and challenges. Value in Health. 2015;18:A530.
15. Chen I, Opiyo N, Tavender E, Mortazhejri S, Rader T, et al. Non‐clinical interventions for reducing unnecessary caesarean section. Cochrane Database of Systematic Reviews. 2018(9).
16. Clyne B, Bradley MC, Hughes C, Fahey T, Lapane KL. Electronic Prescribing and Other Forms of Technology to Reduce Inappropriate Medication Use and Polypharmacy in Older People: A Review of Current Evidence. Clinics in Geriatric Medicine. 2012;28:301-322.
17. Clyne B, Fitzgerald C, Quinlan A, Hardy C, Galvin R, et al. Interventions to Address Potentially Inappropriate Prescribing in Community-Dwelling Older Adults: A Systematic Review of Randomized Controlled Trials. Journal of the American Geriatrics Society. 2016;64(6):1210-1222.
18. Compus COMP, Utilization S. Interventions for appropriate prescribing of proton pump inhibitors: a literature review. Ottawa: Canadian Agency for Drugs and Technologies in Health (CADTH); 2007/02// 2007.
19. Damiani G, Pinnarelli L, Sommella L, Farelli V, Mele L, et al. Appropriateness of fresh-frozen plasma usage in hospital settings: a meta-analysis of the impact of organizational interventions. Transfusion. 2010;50:139-144.
20. Davey P, Marwick CA, Scott CL, Charani E, McNeil K, et al. Interventions to improve antibiotic prescribing practices for hospital inpatients. Cochrane Database of Systematic Reviews. 2017(2).
21. Figueiras A, Sastre I, Gestal-Otero JJ. Effectiveness of educational interventions on the improvement of drug prescription in primary care: a critical literature review. Journal of Evaluation in Clinical Practice. 2001;7:223-241.
22. Fillmore CL, Bray BE, Kawamoto K. Systematic review of clinical decision support interventions with potential for inpatient cost reduction. BMC Medical Informatics & Decision Making. 2013;13:135, 2013.
23. Fleming A, Browne J, Byrne S. The effect of interventions to reduce potentially inappropriate antibiotic prescribing in long-term care facilities: A systematic review of randomised controlled trials. Drugs and Aging. 2013;30:401-408.
24. Flodgren G, Eccles MP, Shepperd S, Scott A, Parmelli E, Beyer FR. An overview of reviews evaluating the effectiveness of financial incentives in changing healthcare professional behaviours and patient outcomes. Cochrane Database of Systematic Reviews. 2011(7).
25. Flodgren G, Hall AM, Goulding L, Eccles MP, Grimshaw JM, et al. Tools developed and disseminated by guideline producers to promote the uptake of their guidelines. Cochrane Database of Systematic Reviews. 2016(8).
26. Flodgren G, O'Brien MA, Parmelli E, Grimshaw JM. Local opinion leaders: effects on professional practice and healthcare outcomes. Cochrane Database of Systematic Reviews. 2019(6).
27. Flodgren G, Rojas‐Reyes MX, Cole N, Foxcroft DR. Effectiveness of organizational infrastructures to promote evidence‐based nursing practice. Cochrane Database of Systematic Reviews. 2012(2).
28. Fønhus MS, Dalsbø TK, Johansen M, Fretheim A, Skirbekk H, Flottorp SA. Patient‐mediated interventions to improve professional practice. Cochrane Database of Systematic Reviews. 2018(9).
29. Forsetlund L, Bjørndal A, Rashidian A, Jamtvedt G, O'Brien MA, et al. Continuing education meetings and workshops: effects on professional practice and health care outcomes. Cochrane Database of Systematic Reviews. 2009(2).
30. Forsetlund L, Eike MC, Gjerberg E, Vist GE. Effect of interventions to reduce potentially inappropriate use of drugs in nursing homes: a systematic review of randomised controlled trials. BMC geriatrics. 2011;11:16.
31. French SD, Green S, Buchbinder R, Barnes H. Interventions for improving the appropriate use of imaging in people with musculoskeletal conditions. Cochrane Database of Systematic Reviews. 2010(1).
32. Giguère A, Légaré F, Grimshaw J, Turcotte S, Fiander M, et al. Printed educational materials: effects on professional practice and healthcare outcomes. Cochrane Database of Systematic Reviews. 2012(10).
33. Gillaizeau F, Chan E, Trinquart L, Colombet I, Walton RT, et al. Computerized advice on drug dosage to improve prescribing practice. Cochrane Database of Systematic Reviews. 2013(11).
34. Green CJ, Maclure M, Fortin PM, Ramsay CR, Aaserud M, Bardal S. Pharmaceutical policies: effects of restrictions on reimbursement. Cochrane Database of Systematic Reviews. 2010(8).
35. Grilli R, Ramsay C, Minozzi S. Mass media interventions: effects on health services utilisation. Cochrane Database of Systematic Reviews. 2002(1).
36. Gurwitz JK, Soumerai SB, Avorn J. Improving medication prescribing and utilization in the nursing home. Journal of the American Geriatrics Society. 1990;38:542-552.
37. Holland R, Desborough J, Goodyer L, Hall S, Wright D, Loke YK. Does pharmacist-led medication review help to reduce hospital admissions and deaths in older people? A systematic review and meta-analysis. British Journal of Clinical Pharmacology. 2008;65:303-316.
38. Ivers N, Jamtvedt G, Flottorp S, Young JM, Odgaard-Jensen J, et al. Audit and feedback: effects on professional practice and healthcare outcomes. Cochrane Database Syst Rev. 2012;6:CD000259.
39. Johansson T, Abuzahra ME, Keller S, Mann E, Faller B, et al. Impact of strategies to reduce polypharmacy on clinically relevant endpoints: a systematic review and meta-analysis. British Journal of Clinical Pharmacology. 2016;82(2):532-548.
40. Johansson T, Flamm M, Sonnichsen A, Schuler J. Interventions to reduce inappropriate polypharmacy: Implications for research and practice. Maturitas. 2017;97:66-68.
41. Kaur S, Mitchell G, Vitetta L, Roberts MS, Gallagher P. Interventions that can Reduce Inappropriate Prescribing in the Elderly: A Systematic Review. Drugs and Aging. 2009;26:1013-1028.
42. Khalil H, Bell B, Chambers H, Sheikh A, Avery AJ. Professional, structural and organizational interventions in primary care for reducing medication errors. Cochrane Database of Systematic Reviews. 2017(10).
43. Lainer M, Mann E, Sonnichsen A. Information technology interventions to improve medication safety in primary care: A systematic review. International Journal for Quality in Health Care. 2013;25:590-598.
44. Loganathan M, Singh S, Franklin BD, Bottle A, Majeed A. Interventions to optimise prescribing in care homes: systematic review (DARE Abstract). Age and Ageing. 2011;40:150-162.
45. Marcum ZA, Handler SM, Wright R, Hanlon JT. Interventions to improve suboptimal prescribing in nursing homes: A narrative review. Am J Geriatr Pharmacother. 2010;8:183-200.
46. Meddings J, Rogers MA, Macy M, Saint S. Systematic review and meta-analysis: reminder systems to reduce catheter-associated urinary tract infections and urinary catheter use in hospitalized patients. Clinical Infectious Diseases. 2010;51:550-560.
47. Niven DJ, Mrklas KJ, Holodinsky JK, Straus SE, Hemmelgarn BR, et al. Towards understanding the de-adoption of low-value clinical practices: A scoping review. BMC Medicine. 2015;13.
48. Nkansah N, Mostovetsky O, Yu C, Chheng T, Beney J, et al. Effect of outpatient pharmacists' non‐dispensing roles on patient outcomes and prescribing patterns. Cochrane Database of Systematic Reviews. 2010(7).
49. O'Brien MA, Rogers S, Jamtvedt G, Oxman AD, Odgaard‐Jensen J, et al. Educational outreach visits: effects on professional practice and health care outcomes. Cochrane Database of Systematic Reviews. 2007(4).
50. Ostini R, Hegney D, Jackson C, Williamson M, Mackson JM, et al. Systematic review of interventions to improve prescribing. Annals of Pharmacotherapy. 2009;43:502-513.
51. Pande S, Hiller JE, Nkansah N, Bero L. The effect of pharmacist‐provided non‐dispensing services on patient outcomes, health service utilisation and costs in low‐ and middle‐income countries. Cochrane Database of Systematic Reviews. 2013(2).
52. Parmelli E, Flodgren G, Schaafsma ME, Baillie N, Beyer FR, Eccles MP. The effectiveness of strategies to change organizational culture to improve healthcare performance. Cochrane Database of Systematic Reviews. 2011(1).
53. Patel Sameer J, Larson Elaine L, Kubin Christine J, Saiman L. A review of antimicrobial control strategies in hospitalized and ambulatory pediatric populations. The Pediatric infectious disease journal. 2007;26(6):531-537.
54. Pearson SA, Moxey A, Robertson J, Hains I, Williamson M, et al. Do computerised clinical decision support systems for prescribing change practice? A systematic review of the literature (1990-2007). BMC Health Services Research. 2009;9:154.
55. Ralston S, Comick A, Nichols E, Parker D, Lanter P. Effectiveness of quality improvement in hospitalization for bronchiolitis: a systematic review. Pediatrics. 2014;134(3):571-581.
56. Ranji SR, Steinman MA, Shojania KG, Gonzales R. Interventions to reduce unnecessary antibiotic prescribing: a systematic review and quantitative analysis. Medical Care. 2008;46:847-862.
57. Ranji SR, Steinman MA, Shojania KG, Sundaram V, Lewis R, et al. Closing the Quality Gap: A Critical Analysis of Quality Improvement Strategies (Vol. 4: Antibiotic Prescribing Behavior). Rockville MD: Agency for Healthcare Research and Quality; 2006/01// 2006.
58. Rankin A, Cadogan CA, Patterson SM, Kerse N, Cardwell CR, et al. Interventions to improve the appropriate use of polypharmacy for older people. Cochrane Database of Systematic Reviews. 2018(9).
59. Rashidian A, Omidvari AH, Vali Y, Sturm H, Oxman AD. Pharmaceutical policies: effects of financial incentives for prescribers. Cochrane Database of Systematic Reviews. 2015(8).
60. Redmond P, Grimes TC, McDonnell R, Boland F, Hughes C, Fahey T. Impact of medication reconciliation for improving transitions of care. Cochrane Database of Systematic Reviews. 2018(8).
61. Reeves S, Pelone F, Harrison R, Goldman J, Zwarenstein M. Interprofessional collaboration to improve professional practice and healthcare outcomes. Cochrane Database of Systematic Reviews. 2017(6).
62. Reeves S, Perrier L, Goldman J, Freeth D, Zwarenstein M. Interprofessional education: effects on professional practice and healthcare outcomes. Cochrane Database of Systematic Reviews. 2013(3).
63. Riordan DO, Walsh KA, Galvin R, Sinnott C, Kearney PM, Byrne S. The effect of pharmacist-led interventions in optimising prescribing in older adults in primary care: A systematic review. SAGE Open Medicine. 2016;4:2050312116652568.
64. Roshanov PS, You JJ, Dhaliwal J, Koff D, Mackay JA, et al. Can computerized clinical decision support systems improve practitioners' diagnostic test ordering behavior? A decision-maker-researcher partnership systematic review. Implementation Science. 2011;6:88, 2011.
65. Schuetz P, Chiappa V, Briel M, Greenwald JL. Procalcitonin algorithms for antibiotic therapy decisions: a systematic review of randomized controlled trials and recommendations for clinical algorithms. Arch Intern Med. 2011;171:1322-1331.
66. Scott A, Sivey P, Ait Ouakrim D, Willenberg L, Naccarella L, et al. The effect of financial incentives on the quality of health care provided by primary care physicians. Cochrane Database of Systematic Reviews. 2011(9).
67. Shojania KG, Jennings A, Mayhew A, Ramsay CR, Eccles MP, Grimshaw J. The effects of on‐screen, point of care computer reminders on processes and outcomes of care. Cochrane Database of Systematic Reviews. 2009(3).
68. Solomon DH, Hashimoto H, Daltroy L, Liang MH. Techniques to improve physicians' use of diagnostic tests: a new conceptual framework. JAMA. 1998;280(23):2020-2027.
69. Soumerai SB, Avorn J. Principles of educational outreach ('academic detailing') to improve clinical decision making. Journal of the American Medical Association. 1990;263:549-556.
70. Steinman Michael A, Ranji Sumant R, Shojania Kaveh G, Gonzales R. Improving antibiotic selection: a systematic review and quantitative analysis of quality improvement strategies. Medical Care. 2006;44(7):617-628.
71. Thompson Coon J, Abbott R, Rogers M, Whear R, Pearson S, et al. Interventions to Reduce Inappropriate Prescribing of Antipsychotic Medications in People With Dementia Resident in Care Homes: A Systematic Review. Journal of the American Medical Directors Association. 2014;15:706-718.
72. Tinmouth A, Macdougall L, Fergusson D, Amin M, Graham ID, et al. Reducing the amount of blood transfused: a systematic review of behavioral interventions to change physicians' transfusion practices. Archives of internal medicine. 2005;165(8):845-852.
73. Tjia J, Velten SJ, Parsons C, Valluri S, Briesacher BA. Studies to reduce unnecessary medication use in frail older adults: a systematic review. Drugs & Aging. 2013;30:285-307.
74. Tzortziou Brown V, Underwood M, Mohamed N, Westwood O, Morrissey D. Professional interventions for general practitioners on the management of musculoskeletal conditions. Cochrane Database of Systematic Reviews. 2016(5).
75. Verbrugghe S. Initiatives to optimize the utilization of laboratory tests. Ottawa: Canadian Agency for Drugs and Technologies in Health; 2014/12// 2014.
76. Vodicka TA, Thompson M, Lucas P, Heneghan C, Blair PS, et al. Reducing antibiotic prescribing for children with respiratory tract infections in primary care: a systematic review. British Journal of General Practice. 2013;63:e445-e454.
77. Wilson K, MacDougall L, Fergusson D, Graham I, Tinmouth A, Hebert PC. The effectiveness of interventions to reduce physician's levels of inappropriate transfusion: what can be learned from a systematic review of the literature. Transfusion. 2002;42:1224-1229.

### Websites

- Canadian Agency for Drugs and Technologies in Health - [https://www.cadth.ca](https://www.cadth.ca/)
- Agency for Healthcare Research and Quality - <http://www.ahrq.gov/>
- Right Care - <https://www.england.nhs.uk/rightcare/> (previously: <http://www.rightcare.nhs.uk/>)
- National Institute for Health and Care Excellence - <https://www.nice.org.uk/>
- Choosing Wisely - <http://www.choosingwisely.org/>
- Kingsfund - <https://www.kingsfund.org.uk/>
- Nuffield Trust - <http://www.nuffieldtrust.org.uk/>

## Appendix 2 - Classification of interventions

Based on taxonomy provided by the Cochrane Effective Practice and Organization of Care (EPOC) Group

| **Educational meetings** | Courses, workshops, conferences or other educational meetings  Including ***educational outreach visits*** (personal visits by a trained person to health workers in their own settings, to provide information with the aim of changing practice) and ***inter-professional education*** (continuing education for health professionals that involves more than one profession in joint, interactive learning) |
| --- | --- |
| Educational materials | Distribution to individuals, or groups, of educational materials to support clinical care, i.e., any intervention in which knowledge is distributed. For example this may be facilitated by the internet, learning critical appraisal skills; skills for electronic retrieval of information, diagnostic formulation; question formulation |
| Reminders | Manual or computerized interventions that prompt health workers to perform an action during a consultation with a patient, for example computer ***decision support systems***. |
| Audit and feedback | A summary of health workers’ performance over a specified period of time, given to them in a written, electronic or verbal format. The summary may include recommendations for clinical action. |
| Patient directed interventions | Interventions aimed at patients; e.g. patient information, posters in waiting room, mass media campaign. |
| Organizational interventions | Interventions aimed at a group of professionals, interprovider relations, organization or institution  Examples:   - Revision of professional roles: ‘professional substitution’, ‘boundary encroachment’; includes the shifting of roles among health professionals. For example, nurse midwives providing obstetrical care; pharmacists providing drug counselling that was formerly provided by nurses and physicians; nutritionists providing nursing care; physical therapists providing nursing care. Also includes expansion of role to include new tasks. - Clinical multidisciplinary teams: creation of a new team of health professionals of different disciplines or additions of new members to the team who work together to care for patients - Formal integration of services across sectors or teams or the organization of services to bring all services together at one time also sometimes called ‘seamless care’ - Skill mix changes: changes in numbers, types or qualifications of staff - Local opinion leaders: the identification and use of identifiable local opinion leaders to promote good clinical practice. - Continuity of care: including one or many episodes of care for inpatients or outpatients)   - Arrangements for follow-up.   - Case management (including co-ordination of assessment, treatment and arrangement for referrals - Communication and case discussion between distant health professionals e.g. telephone links; telemedicine; there is a television/video link between specialist and remote nurse practitioners - Continuous quality improvement: an iterative process to review and improve care that includes involvement of healthcare teams, analysis of a process or system, a structured process improvement method or problem solving approach, and use of data analysis to assess changes - Clinical Practice Guidelines: clinical guidelines are systematically developed statements to assist healthcare providers and patients to decide on appropriate health care for specific clinical circumstances'(US IOM). - Clinical incident reporting: system for reporting critical incidents, - Routine patient-reported outcome measures: routine administration and reporting of patient-reported outcome measures to providers and/or patients - Local consensus processes: formal or informal local consensus processes, for example agreeing a clinical protocol to manage a patient group, adapting a guideline for a local health system or promoting the implementation of guidelines. |
| Structural interventions | - Changes to the setting/site of service delivery e.g. moving a family planning service from a hospital to a school - Ownership, accreditation, and affiliation status of hospitals and other facilities |
| Regulatory interventions | Any intervention that aims to change health services delivery by regulation or law  Examples:   - Changes in medical liability - Licensure |
| Financial interventions | Any financial interventions aimed at either healthcare professional, patient, health care system |

1. Grol R, Wensing M, Eccles MP, Davis D. *Improving patient care: the implementation of change in health care.*: John Wiley & Sons; 2013.

2. Effective Practice and Organisation of Care (EPOC); EPOC Taxonomy. *Available at:* [*https://epoccochraneorg/epoc-taxonomy*](https://epoccochraneorg/epoc-taxonomy)*.* 2015.

3. *Effective Practice and Organisation of Care (EPOC); The EPOC taxonomy of health systems interventions. EPOC Resources for review authors.* Oslo: Norwegian Knowledge Centre for the Health Services;2016.

4. Higgins JP, Altman DG, Gotzsche PC, Juni P, Moher D, et al. The Cochrane Collaboration's tool for assessing risk of bias in randomised trials. *BMJ.* 2011;343:d5928.

5. Higgins J, Green S, (editors). Cochrane Handbook for Systematic Reviews of Interventions Version 5.1.0 [updated March 2011]. In: The Cochrane Collaboration; 2011: <http://handbook-5-1.cochrane.org/>.

6. Bolzern J, Mnyama N, Bosanquet K, Torgerson DJ. A review of cluster randomized trials found statistical evidence of selection bias. *J Clin Epidemiol.* 2018;99:106-112.

7. Brierley G, Brabyn S, Torgerson D, Watson J. Bias in recruitment to cluster randomized trials: a review of recent publications. *J Eval Clin Pract.* 2012;18(4):878-886.

8. Puffer S, Torgerson D, Watson J. Evidence for risk of bias in cluster randomised trials: review of recent trials published in three general medical journals. *BMJ.* 2003;327(7418):785-789.

9. Allard J, Hébert R, Rioux M, Asselin J, Voyer L. Efficacy of a clinical medication review on the number of potentially inappropriate prescriptions prescribed for community-dwelling elderly people. *CMAJ.* 2001;164(9):1291-1296.

10. Althabe F, Belizán JM, Villar J, Alexander S, Bergel E, et al. Mandatory second opinion to reduce rates of unnecessary caesarean sections in Latin America: a cluster randomised controlled trial. *Lancet.* 2004;363(9425):1934-1940.

11. Althabe F, Buekens P, Bergel E, Belizán JM, Campbell MK, et al. A behavioral intervention to improve obstetrical care. *N Engl J Med.* 2008;358(18):1929-1940.

12. Altiner A, Brockmann S, Sielk M, Wilm S, Wegscheider K, Abholz HH. Reducing antibiotic prescriptions for acute cough by motivating GPs to change their attitudes to communication and empowering patients: a cluster-randomized intervention study. *J Antimicrob Chemother.* 2007;60(3):638-644.

13. Anderson JF, McEwan KL, Hrudey WP. Effectiveness of notification and group education in modifying prescribing of regulated analgesics. *CMAJ.* 1996;154(1):31-39.

14. Awad AI, Eltayeb IB, Baraka OZ. Changing antibiotics prescribing practices in health centers of Khartoum State, Sudan. *Eur J Clin Pharmacol.* 2006;62(2):135-142.

15. Baker R, Falconer Smith J, Lambert Paul C. Randomised controlled trial of the effectiveness of feedback in improving test ordering in general practice. *Scand J Prim Health Care.* 2003;21(4):219-223.

16. Bates DW, Kuperman GJ, Rittenberg E, Teich JM, Fiskio J, et al. A randomized trial of a computer-based intervention to reduce utilization of redundant laboratory tests. *Am J Med.* 1999;106(2):144-150.

17. Bearcroft PW, Small JH, Flower CD. Chest radiography guidelines for general practitioners: a practical approach. *Clin Radiol.* 1994;49(1):56-58.

18. Berings D, Blondeel L, Habraken H. The effect of industry-independent drug information on the prescribing of benzodiazepines in general practice. *Eur J Clin Pharmacol.* 1995;46(6):501-505.

19. Bhatia RS, Dudzinski DM, Malhotra R, Milford CE, Sanborn DMY, et al. Educational intervention to reduce outpatient inappropriate echocardiograms: A randomized control trial. *JACC Cardiovasc Imaging.* 2014;7:857-866.

20. Bhatia RS, Ivers NM, Yin XC, Myers D, Nesbitt GC, et al. Improving the Appropriate Use of Transthoracic Echocardiography: The Echo WISELY Trial. *J Am Coll Cardiol.* 2017;70(9):1135-1144.

21. Boersma MN, Huibers CJA, Drenth-van Maanen AC, Emmelot-Vonk MH, Wilting I, Knol W. The effect of providing prescribing recommendations on appropriate prescribing: A cluster-randomized controlled trial in older adults in a preoperative setting. *British Journal of Clinical Pharmacology.* 2019;85(9):1974-1983.

22. Bourgeois FC, Linder J, Johnson SA, Co JP, Fiskio J, Ferris TG. Impact of a computerized template on antibiotic prescribing for acute respiratory infections in children and adolescents. *Clinical pediatrics.* 2010;49(10):976-983.

23. Braybrook S, Walker R. Influencing prescribing in primary care: a comparison of two different prescribing feedback methods. *J Clin Pharm Ther.* 1996;21(4):247-254.

24. Braybrook S, Walker R. Influencing NSAID prescribing in primary care using different feedback strategies. *Pharmacy world & science : PWS.* 2000;22(2):39-46.

25. Bregnhøj L, Thirstrup S, Kristensen MB, Bjerrum L, Sonne J. Combined intervention programme reduces inappropriate prescribing in elderly patients exposed to polypharmacy in primary care. *Eur J Clin Pharmacol.* 2009;65(2):199-207.

26. Briel M, Langewitz W, Tschudi P, Young J, Hugenschmidt C, Bucher Heiner C. Communication training and antibiotic use in acute respiratory tract infections. A cluster randomised controlled trial in general practice. *Swiss medical weekly.* 2006;136(15-16):241-247.

27. Bryant LJ, Coster G, Gamble GD, McCormick RN. The General Practitioner-Pharmacist Collaboration (GPPC) study: a randomised controlled trial of clinical medication reviews in community pharmacy. *The International journal of pharmacy practice.* 2011;19(2):94-105.

28. Campins L, Serra-Prat M, Gozalo I, Lopez D, Palomera E, et al. Randomized controlled trial of an intervention to improve drug appropriateness in community-dwelling polymedicated elderly people. *Fam Pract.* 2017;34(1):36-42.

29. Cánovas JJ, Hernández PJ, Botella JJ. Effectiveness of internal quality assurance programmes in improving clinical practice and reducing costs. *J Eval Clin Pract.* 2009;15(5):813-819.

30. Carney PA, Abraham L, Cook A, Feig SA, Sickles EA, et al. Impact of an educational intervention designed to reduce unnecessary recall during screening mammography. *Acad Radiol.* 2012;19:1114-1120.

31. Christakis DA, Zimmerman FJ, Wright JA, Garrison MM, Rivara FP, Davis RL. A randomized controlled trial of point-of-care evidence to improve the antibiotic prescribing practices for otitis media in children. *Pediatrics.* 2001;107(2):E15.

32. Clyne B, Smith SM, Hughes CM, Boland F, Bradley MC, et al. Effectiveness of a Multifaceted Intervention for Potentially Inappropriate Prescribing in Older Patients in Primary Care: A Cluster-Randomized Controlled Trial (OPTI-SCRIPT Study). *Ann Fam Med.* 2015;13(6):545-553.

33. Clyne B, Smith SM, Hughes CM, Boland F, Cooper JA, Fahey T. Sustained effectiveness of a multifaceted intervention to reduce potentially inappropriate prescribing in older patients in primary care (OPTI-SCRIPT study). *Implement Sci.* 2016;11(1):79.

34. Coenen S, Van Royen P, Michiels B, Denekens J. Optimizing antibiotic prescribing for acute cough in general practice: a cluster-randomized controlled trial. *J Antimicrob Chemother.* 2004;54(3):661-672.

35. Crotty M, Halbert J, Rowett D, Giles L, Birks R, et al. An outreach geriatric medication advisory service in residential aged care: a randomised controlled trial of case conferencing. *Age Ageing.* 2004;33(6):612-617.

36. Culbertson VL, Force RW, Cady PS, Force WS. Positive impact of a follow-up phone call to community pharmacies in a medicaid drug utilization program. *Ann Pharmacother.* 1999;33(5):541-547.

37. Daley P, Garcia D, Inayatullah R, Penney C, Boyd S. Modified Reporting of Positive Urine Cultures to Reduce Inappropriate Treatment of Asymptomatic Bacteriuria Among Nonpregnant, Noncatheterized Inpatients: A Randomized Controlled Trial. *Infect Control Hosp Epidemiol.* 2018;39(7):814-819.

38. Davies B, Hodnett E, Hannah M, O'Brien-Pallas L, Pringle D, et al. Fetal health surveillance: a community-wide approach versus a tailored intervention for the implementation of clinical practice guidelines. *CMAJ.* 2002;167(5):469-474.

39. de Burgh, Mant A, Mattick RP, Donnelly N, Hall W, Bridges-Webb C. A controlled trial of educational visiting to improve benzodiazepine prescribing in general practice. *Aust J Public Health.* 1995;19:142-148.

40. Dey P, Simpson CW, Collins SI, Hodgson G, Dowrick CF, et al. Implementation of RCGP guidelines for acute low back pain: a cluster randomised controlled trial. *Br J Gen Pract.* 2004;54(498):33-37.

41. Dolovich L, Levine M, Tarajos R, Duku E. Promoting Optimal Antibiotic Therapy for Otitis Media Using Commercially Sponsored Evidence-Based Detailing: A Prospective Controlled Trial. *Drug Information Journal.* 1999;33(4):1067-1077.

42. Doyne EO, Alfaro MP, Siegel RM, Atherton HD, Schoettker PJ, et al. A randomized controlled trial to change antibiotic prescribing patterns in a community. *Archives of pediatrics & adolescent medicine.* 2004;158(6):577-583.

43. Dranitsaris G, Spizzirri D, Pitre M, McGeer A. A randomized trial to measure the optimal role of the pharmacist in promoting evidence-based antibiotic use in acute care hospitals. *International journal of technology assessment in health care.* 2001;17(2):171-180.

44. Eccles M, Steen N, Grimshaw J, Thomas L, McNamee P, et al. Effect of audit and feedback, and reminder messages on primary-care radiology referrals: a randomised trial. *Lancet.* 2001;357(9266):1406-1409.

45. Eltayeb IB, Awad AI, Mohamed-Salih MS, Daffa-Alla MA, Ahmed MB, et al. Changing the prescribing patterns of sexually transmitted infections in the White Nile Region of Sudan. *Sexually transmitted infections.* 2005;81(5):426-427.

46. Engers AJ, Wensing M, van Tulder MW, Timmermans A, Oostendorp RA, et al. Implementation of the Dutch low back pain guideline for general practitioners: a cluster randomized controlled trial. *Spine.* 2005;30(6):559-600.

47. Fender GR, Prentice A, Gorst T, Nixon RM, Duffy SW, et al. Randomised controlled trial of educational package on management of menorrhagia in primary care: the Anglia menorrhagia education study. *BMJ.* 1999;318(7193):1246-1250.

48. Fenton JJ, Kravitz RL, Jerant A, Paterniti DA, Bang H, et al. Promoting Patient-Centered Counseling to Reduce Use of Low-Value Diagnostic Tests: A Randomized Clinical Trial. *JAMA Intern Med.* 2016;176(2):191-197.

49. May L, Franks P, Jerant A, Fenton J. Watchful Waiting Strategy May Reduce Low-Value Diagnostic Testing. *JABFM.* 2016;29(6):710-717.

50. Field TS, Rochon P, Lee M, Gavendo L, Baril JL, Gurwitz JH. Computerized clinical decision support during medication ordering for long-term care residents with renal insufficiency. *Journal of the American Medical Informatics Association : JAMIA.* 2009;16(4):480-485.

51. Fine MJ, Stone RA, Lave JR, Hough LJ, Obrosky DS, et al. Implementation of an evidence-based guideline to reduce duration of intravenous antibiotic therapy and length of stay for patients hospitalized with community-acquired pneumonia: a randomized controlled trial. *Am J Med.* 2003;115(5):343-351.

52. Finkelstein JA, Davis RL, Dowell SF, Metlay JP, Soumerai SB, et al. Reducing antibiotic use in children: a randomized trial in 12 practices. *Pediatrics.* 2001;108:1-7.

53. Finkelstein JA, Huang SS, Kleinman K, Rifas-Shiman SL, Stille CJ, et al. Impact of a 16-community trial to promote judicious antibiotic use in Massachusetts. *Pediatrics.* 2008;121(1):e15-23.

54. Flottorp S, Oxman AD, Havelsrud K, Treweek S, Herrin J. Cluster randomised controlled trial of tailored interventions to improve the management of urinary tract infections in women and sore throat. *BMJ.* 2002;325(7360):367.

55. Fossey J, Ballard C, Juszczak E, James I, Alder N, et al. Effect of enhanced psychosocial care on antipsychotic use in nursing home residents with severe dementia: cluster randomised trial. *BMJ.* 2006;332(7544):756-761.

56. Francis NA, Butler CC, Hood K, Simpson S, Wood F, Nuttall J. Effect of using an interactive booklet about childhood respiratory tract infections in primary care consultations on reconsulting and antibiotic prescribing: a cluster randomised controlled trial. *BMJ.* 2009;339:b2885.

57. Frankenthal D, Lerman Y, Kalendaryev E, Lerman Y. Intervention with the screening tool of older persons potentially inappropriate prescriptions/screening tool to alert doctors to right treatment criteria in elderly residents of a chronic geriatric facility: a randomized clinical trial. *J Am Geriatr Soc.* 2014;62(9):1658-1665.

58. French SD, McKenzie JE, O'Connor DA, Grimshaw JM, Mortimer D, et al. Evaluation of a theory-informed implementation intervention for the management of acute low back pain in general medical practice: the IMPLEMENT cluster randomised trial. *PLoS One.* 2013;8(6):e65471.

59. Gallagher PF, O'Connor MN, O'Mahony D. Prevention of potentially inappropriate prescribing for elderly patients: a randomized controlled trial using STOPP/START criteria. *Clinical pharmacology and therapeutics.* 2011;89(6):845-854.

60. Garcia-Gollarte F, Baleriola-Julvez J, Ferrero-Lopez I, Cuenllas-Diaz A, Cruz-Jentoft AJ. An educational intervention on drug use in nursing homes improves health outcomes resource utilization and reduces inappropriate drug prescription. *J Am Med Dir Assoc.* 2014;15(12):885-891.

61. Gjelstad S, Høye S, Straand J, Brekke M, Dalen I, Lindbæk M. Improving antibiotic prescribing in acute respiratory tract infections: cluster randomised trial from Norwegian general practice (prescription peer academic detailing (Rx-PAD) study). *BMJ.* 2013;347:f4403-f4403.

62. Goldberg HI, Deyo RA, Taylor VM, Cheadle AD, Conrad DA, et al. Can evidence change the rate of back surgery? A randomized trial of community-based education. *Effective clinical practice : ECP.* 2001;4(3):95-104.

63. Hadiyono JE, Suryawati S, Danu SS, Sunartono N, Santoso B. Interactional group discussion: results of a controlled trial using a behavioral intervention to reduce the use of injections in public health facilities. *Social science & medicine (1982).* 1996;42(8):1177-1183.

64. Hemminki E, Heikkilä K, Sevón T, Koponen P. Special features of health services and register based trials - experiences from a randomized trial of childbirth classes. *BMC Health Serv Res.* 2008;8:126.

65. Hodnett ED, Kaufman K, O'Brien-Pallas L, Chipman M, Watson-MacDonell J, Hunsburger W. A strategy to promote research-based nursing care: effects on childbirth outcomes. *Research in nursing & health.* 1996;19(1):13-20.

66. Holm M. Intervention against long-term use of hypnotics/sedatives in general practice. *Scand J Prim Health Care.* 1990;8(2):113-117.

67. Ilett KF, Johnson S, Greenhill G, Mullen L, Brockis J, et al. Modification of general practitioner prescribing of antibiotics by use of a therapeutics adviser (academic detailer). *British journal of clinical pharmacology.* 2000;49(2):168-173.

68. Kerry S, Oakeshott P, Dundas D, Williams J. Influence of postal distribution of the Royal College of Radiologists' guidelines, together with feedback on radiological referral rates, on X-ray referrals from general practice: a randomized controlled trial. *Family practice.* 2000;17(1):46-52.

69. Kirkham J, Maxwell C, Velkers C, Leung R, Moffat K, Seitz D. Optimizing Prescribing of Antipsychotics in Long-Term Care (OPAL): A Stepped-Wedge Trial. *J Am Med Dir Assoc.* 2019;23:23.

70. Lagerløv P, Loeb M, Andrew M, Hjortdahl P. Improving doctors' prescribing behaviour through reflection on guidelines and prescription feedback: a randomised controlled study. *Quality in health care : QHC.* 2000;9(3):159-165.

71. Lemiengre MB, Verbakel JY, Colman R, De Burghgraeve T, Buntinx F, et al. Reducing inappropriate antibiotic prescribing for children in primary care: a cluster randomised controlled trial of two interventions. *Br J Gen Pract.* 2018;68(668):e204-e210.

72. Linder JA, Schnipper JL, Tsurikova R, Yu T, Volk LA, et al. Documentation-based clinical decision support to improve antibiotic prescribing for acute respiratory infections in primary care: a cluster randomised controlled trial. *Inform Prim Care.* 2009;17(4):231-240.

73. Loeb M, Brazil K, Lohfeld L, McGeer A, Simor A, et al. Effect of a multifaceted intervention on number of antimicrobial prescriptions for suspected urinary tract infections in residents of nursing homes: cluster randomised controlled trial. *BMJ.* 2005;331:669.

74. Lundborg CS, Wahlström R, Oke T, Tomson G, Diwan VK. Influencing prescribing for urinary tract infection and asthma in primary care in Sweden: a randomized controlled trial of an interactive educational intervention. *J Clin Epidemiol.* 1999;52(8):801-812.

75. Macfarlane J, Holmes W, Gard P, Thornhill D, Macfarlane R, Hubbard R. Reducing antibiotic use for acute bronchitis in primary care: blinded, randomised controlled trial of patient information leaflet. *BMJ.* 2002;324(7329):91-94.

76. Mainous Iii AG, Hueston WJ, Love MM, Evans ME, Finger R. An evaluation of statewide strategies to reduce antibiotic overuse. *Fam Med.* 2000;32:22-29.

77. Martens JD, van der Weijden T, Severens JL, de Clercq PA, de Bruijn DP, et al. The effect of computer reminders on GPs' prescribing behaviour: a cluster-randomised trial. *Int J Med Inform.* 2007;76:S403-S416.

78. McIsaac WJ, Goel V, To T, Permaul JA, Low DE. Effect on antibiotic prescribing of repeated clinical prompts to use a sore throat score: lessons from a failed community intervention study. *The Journal of family practice.* 2002;51(4):339-344.

79. Meador KG, Taylor JA, Thapa PB, Fought RL, Ray WA. Predictors of antipsychotic withdrawal or dose reduction in a randomized controlled trial of provider education. *J Am Geriatr Soc.* 1997;45(2):207-210.

80. Meeker D, Knight TK, Friedberg MW, Linder JA, Goldstein NJ, et al. Nudging guideline-concordant antibiotic prescribing: A randomized clinical trial. *JAMA Intern Med.* 2014;174:425-431.

81. Meeker D, Linder JA, Fox CR, Friedberg MW, Persell SD, et al. Effect of Behavioral Interventions on Inappropriate Antibiotic Prescribing Among Primary Care Practices: A Randomized Clinical Trial. *JAMA.* 2016;315(6):562-570.

82. Metlay JP, Camargo, Jr., C A, MacKenzie T, McCulloch C, et al. Cluster-Randomized Trial to Improve Antibiotic Use for Adults With Acute Respiratory Infections Treated in Emergency Departments. *Ann Emerg Med.* 2007;50:221-230.

83. Milos V, Rekman E, Bondesson Å, Eriksson T, Jakobsson U, et al. Improving the quality of pharmacotherapy in elderly primary care patients through medication reviews: a randomised controlled study. *Drugs Aging.* 2013;30(4):235-246.

84. Monette J, Miller MA, Monette M, Laurier C, Boivin JF, et al. Effect of an educational intervention on optimizing antibiotic prescribing in long-term care facilities. *J Am Geriatr Soc.* 2007;55:1231-1235.

85. Nilsson G, Hjemdahl P, Hässler A, Vitols S, Wallén NH, Krakau I. Feedback on prescribing rate combined with problem-oriented pharmacotherapy education as a model to improve prescribing behaviour among general practitioners. *Eur J Clin Pharmacol.* 2001;56(11):843-848.

86. Oakeshott P, Kerry SM, Williams JE. Randomized controlled trial of the effect of the Royal College of Radiologists' guidelines on general practitioners' referrals for radiographic examination. *Br J Gen Pract.* 1994;44(382):197-200.

87. Pagaiya N, Garner P. Primary care nurses using guidelines in Thailand: a randomized controlled trial. *Tropical medicine & international health : TM & IH.* 2005;10(5):471-477.

88. Patterson SM, Hughes CM, Crealey G, Cardwell C, Lapane KL. An evaluation of an adapted U.S. model of pharmaceutical care to improve psychoactive prescribing for nursing home residents in northern ireland (fleetwood northern ireland study). *J Am Geriatr Soc.* 2010;58(1):44-53.

89. Payne SMC, Ash A, Restuccia JD. The role of feedback in reducing medically unnecessary hospital use. *Med Care.* 1991;29:91-106.

90. Persell SD, Doctor JN, Friedberg MW, Meeker D, Friesema E, et al. Behavioral interventions to reduce inappropriate antibiotic prescribing: a randomized pilot trial. *BMC Infect Dis.* 2016;16:373.

91. Pettersson E, Vernby A, Molstad S, Lundborg CS. Can a multifaceted educational intervention targeting both nurses and physicians change the prescribing of antibiotics to nursing home residents? A cluster randomized controlled trial. *J Antimicrob Chemother.* 2011;66:2659-2666.

92. Pimlott NJG, Hux JE, Wilson LM, Kahan M, Li C, Rosser WW. Educating physicians to reduce benzodiazepine use by elderly patients: a randomized controlled trial. *CMAJ.* 2003;168(7):835-839.

93. Pit SW, Byles JE, Henry DA, Holt L, Hansen V, Bowman DA. A Quality Use of Medicines program for general practitioners and older people: a cluster randomised controlled trial. *Med J Aust.* 2007;187(1):23-30.

94. Pitkälä KH, Juola AL, Kautiainen H, Soini H, Finne-Soveri UH, et al. Education to reduce potentially harmful medication use among residents of assisted living facilities: a randomized controlled trial. *J Am Med Dir Assoc.* 2014;15(12):892-898.

95. Pshetizky Y, Naimer S, Shvartzman P. Acute otitis media--a brief explanation to parents and antibiotic use. *Family practice.* 2003;20(4):417-419.

96. Raebel MA, Charles J, Dugan J, Carroll NM, Korner EJ, et al. Randomized trial to improve prescribing safety in ambulatory elderly patients. *J Am Geriatr Soc.* 2007;55(7):977-985.

97. Regev-Yochay G, Raz M, Dagan R, Roizin H, Morag B, et al. Reduction in antibiotic use following a cluster randomized controlled multifaceted intervention: the Israeli judicious antibiotic prescription study. *Clin Infect Dis.* 2011;53(1):33-41.

98. Rognstad S, Brekke M, Fetveit A, Dalen I, Straand J. Prescription peer academic detailing to reduce inappropriate prescribing for older patients: A cluster randomised controlled trial. *Br J Gen Pract.* 2013;63:e554-e562.

99. Rothschild JM, McGurk S, Honour M, Lu L, McClendon AA, et al. Assessment of education and computerized decision support interventions for improving transfusion practice. *Transfusion.* 2007;47(2):228-239.

100. Rubin GL, Schofield WN, Dean MG, Shakeshaft AP. Appropriateness of red blood cell transfusions in major urban hospitals and effectiveness of an intervention. *Med J Aust.* 2001;175(7):354-358.

101. Samore MH, Bateman K, Alder SC, Hannah E, Donnelly S, et al. Clinical decision support and appropriateness of antimicrobial prescribing: A randomized trial. *JAMA.* 2005;294:2305-2314.

102. Santoso B, Suryawati S, Prawaitasari JE. Small group intervention vs formal seminar for improving appropriate drug use. *Social science & medicine (1982).* 1996;42(8):1163-1168.

103. Schectman JM, Schroth WS, Verme D, Voss JD. Randomized controlled trial of education and feedback for implementation of guidelines for acute low back pain. *J Gen Intern Med.* 2003;18(10):773-780.

104. Schmidt I, Claesson CB, Westerholm B, Nilsson LG, Svarstad BL. The impact of regular multidisciplinary team interventions on psychotropic prescribing in Swedish nursing homes. *J Am Geriatr Soc.* 1998;46(1):77-82.

105. Seager JM, Howell-Jones RS, Dunstan FD, Lewis MAO, Richmond S, Thomas DW. A randomised controlled trial of clinical outreach education to rationalise antibiotic prescribing for acute dental pain in the primary care setting. *Br Dent J.* 2005;201(4):217-222; discussion 216.

106. Shojania KG, Yokoe D, Platt R, Fiskio J, Ma'luf N, Bates DW. Reducing vancomycin use utilizing a computer guideline: results of a randomized controlled trial. *J Am Med Inform Assoc.* 1998;5:554-562.

107. Simon SR, Smith DH, Feldstein AC, Perrin N, Yang X, et al. Computerized prescribing alerts and group academic detailing to reduce the use of potentially inappropriate medications in older people. *J Am Geriatr Soc.* 2006;54:963-968.

108. Solomon DH, Van Houten L, Glynn RJ, Baden L, Curtis K, et al. Academic detailing to improve use of broad-spectrum antibiotics at an academic medical center. *Arch Intern Med.* 2001;161(15):1897-1902.

109. Søndergaard J, Andersen M, Støvring H, Kragstrup J. Mailed prescriber feedback in addition to a clinical guideline has no impact: a randomised, controlled trial. *Scand J Prim Health Care.* 2003;21(1):47-51.

110. Soumerai SB, McLaughlin TJ, Gurwitz JH, Guadagnoli E, Hauptman PJ, et al. Effect of local medical opinion leaders on quality of care for acute myocardial infarction: a randomized controlled trial. *JAMA : the journal of the American Medical Association.* 1998;279(17):1358-1363.

111. Spinewine A, Swine C, Dhillon S, Lambert P, Nachega JB, et al. Effect of a collaborative approach on the quality of prescribing for geriatric inpatients: a randomized, controlled trial. *J Am Geriatr Soc.* 2007;55(5):658-665.

112. Tamblyn R, Huang A, Perreault R, Jacques A, Roy D, et al. The medical office of the 21st century (MOXXI): effectiveness of computerized decision-making support in reducing inappropriate prescribing in primary care. *CMAJ.* 2003;169(6):549-556.

113. Taylor J, A., Kwan-Gett TSC, McMahon EM. Effectiveness of a parental educational intervention in reducing antibiotic use in children: a randomized controlled trial. *Pediatr Infect Dis J.* 2005;24(6):489-493.

114. Terrell KM, Perkins AJ, Dexter PR, Hui SL, Callahan CM, Miller DK. Computerized decision support to reduce potentially inappropriate prescribing to older emergency department patients: a randomized, controlled trial. *J Am Geriatr Soc.* 2009;57(8):1388-1394.

115. Thomas RE, Croal BL, Ramsay C, Eccles M, Grimshaw J. Effect of enhanced feedback and brief educational reminder messages on laboratory test requesting in primary care: a cluster randomised trial. *Lancet.* 2006;367(9527):1990-1996.

116. Tierney WM, Miller ME, McDonald CJ. The effect on test ordering of informing physicians of the charges for outpatient diagnostic tests. *N Engl J Med.* 1990;322(21):1499-1504.

117. Trietsch J, van Steenkiste B, Grol R, Winkens B, Ulenkate H, et al. Effect of audit and feedback with peer review on general practitioners' prescribing and test ordering performance: a cluster-randomized controlled trial. *BMC Fam Pract.* 2017;18(1):53.

118. Urbiztondo I, Bjerrum L, Caballero L, Suarez MA, Olinisky M, Cordoba G. Decreasing Inappropriate Use of Antibiotics in Primary Care in Four Countries in South America-Cluster Randomized Controlled Trial. *Antibiotics (Basel).* 2017;6(4).

119. van Driel M, L., Coenen S, Dirven K, Lobbestael J, Janssens I, et al. What is the role of quality circles in strategies to optimise antibiotic prescribing? A pragmatic cluster-randomised controlled trial in primary care. *Qual Saf Health Care.* 2007;16(3):197-202.

120. van Eijk M, E., Avorn J, Porsius AJ, de Boer. Reducing prescribing of highly anticholinergic antidepressants for elderly people: randomised trial of group versus individual academic detailing. *BMJ.* 2001;322:654-657.

121. van Wijk MA, van der Lei J, Mosseveld M, Bohnen AM, van Bemmel JH. Assessment of decision support for blood test ordering in primary care. a randomized trial. *Ann Intern Med.* 2001;134(4):274-281.

122. Verstappen WHJM, van der Weijden T, Sijbrandij J, Smeele I, Hermsen J, et al. Effect of a practice-based strategy on test ordering performance of primary care physicians: a randomized trial. *JAMA.* 2003;289(18):2407-2412.

123. Verstappen WH, van der Weijden T, Dubois WI, Smeele I, Hermsen J, et al. Improving test ordering in primary care: the added value of a small-group quality improvement strategy compared with classic feedback only. *Ann Fam Med.* 2004;2(6):569-575.

124. Vierhout WP, Knottnerus JA, van OA, Crebolder HF, Pop P, et al. Effectiveness of joint consultation sessions of general practitioners and orthopaedic surgeons for locomotor-system disorders. *Lancet.* 1995;346(8981):990-994.

125. Voorn VMA, Marang-van de Mheen PJ, van der Hout A, Hofstede SN, So-Osman C, et al. The effectiveness of a de-implementation strategy to reduce low-value blood management techniques in primary hip and knee arthroplasty: a pragmatic cluster-randomized controlled trial. *Implement Sci.* 2017;12(1):72.

126. Weber V, White A, McIlvried R. An electronic medical record (EMR)-based intervention to reduce polypharmacy and falls in an ambulatory rural elderly population. *J Gen Intern Med.* 2008;23(4):399-404.

127. Wei X, Zhang Z, Walley JD, Hicks JP, Zeng J, et al. Effect of a training and educational intervention for physicians and caregivers on antibiotic prescribing for upper respiratory tract infections in children at primary care facilities in rural China: a cluster-randomised controlled trial. *Lancet Glob Health.* 2017;5(12):e1258-e1267.

128. Zhang Z, Dawkins B, Hicks JP, Walley JD, Hulme C, et al. Cost-effectiveness analysis of a multi-dimensional intervention to reduce inappropriate antibiotic prescribing for children with upper respiratory tract infections in China. *Tropical Medicine & International Health.* 2018;29:29.

129. Weller D, May F, Rowett D, Esterman A, Pinnock C, et al. Promoting better use of the PSA test in general practice: randomized controlled trial of educational strategies based on outreach visits and mailout. *Family practice.* 2003;20(6):655-661.

130. Welschen I, Kuyvenhoven Marijke M, Hoes Arno W, Verheij Theo JM. Effectiveness of a multiple intervention to reduce antibiotic prescribing for respiratory tract symptoms in primary care: randomised controlled trial. *BMJ.* 2004;329(7463):431.

131. Wilson EJ. Realities of Practice: Development and Implementation of Clinical Practice Guidelines for Acute Respiratory Infections in Young Children. 2002.

132. Wilson EJ, Nasrin D, Dear KB, Douglas RM. Changing GPs' antibiotic prescribing: a randomised controlled trial. *Communicable diseases intelligence.* 2003;27:S32-S38.

133. Winkens RA, Pop P, Bugter-Maessen AM, Grol RP, Kester AD, et al. Randomised controlled trial of routine individual feedback to improve rationality and reduce numbers of test requests. *Lancet.* 1995;345(8948):498-502.

134. Zwar N, Wolk J, Gordon J, Sanson-Fisher R, Kehoe L. Influencing antibiotic prescribing in general practice: a trial of prescriber feedback and management guidelines. *Family practice.* 1999;16(5):495-500.

1. Grol R, Wensing M, Eccles MP, Davis D. *Improving patient care: the implementation of change in health care.* John Wiley & Sons; 2013.

2. Effective Practice and Organisation of Care (EPOC); EPOC Taxonomy. *Available at:* [*https://epoccochraneorg/epoc-taxonomy*](https://epoccochraneorg/epoc-taxonomy). 2015;

3. *Effective Practice and Organisation of Care (EPOC); The EPOC taxonomy of health systems interventions. EPOC Resources for review authors*. Vol. Available at: <http://epoc.cochrane.org/epoc-specific-resources-review-authors>. 2016.

4. Higgins JP, Altman DG, Gotzsche PC, et al. The Cochrane Collaboration's tool for assessing risk of bias in randomised trials. *BMJ (Clinical research ed)*. Oct 18 2011;343:d5928. doi:10.1136/bmj.d5928
